# Bioprospection of actinobacteria derived from freshwater sediments for their potential to produce antimicrobial compounds

**DOI:** 10.1186/s12934-018-0912-0

**Published:** 2018-05-05

**Authors:** Zothanpuia, Ajit Kumar Passari, Vincent Vineeth Leo, Preeti Chandra, Brijesh Kumar, Chandra Nayak, Abeer Hashem, Elsayed Fathi Abd_Allah, Abdulaziz A. Alqarawi, Bhim Pratap Singh

**Affiliations:** 1 Molecular Microbiology and Systematics Laboratory, Department of Biotechnology, Mizoram University, Aizawl, Mizoram 796004 India; 2 SAIF, CSIR-Central Drug Research Institute (CSIR-CDRI), Lucknow, 226012 India; 3 University of Mysore, Manasagangotri, Mysore India; 4 Botany and Microbiology Department, College of Science, King Saud University, P.O. Box. 2460, Riyadh, 11451 Saudi Arabia; 5 Mycology and Plant Disease Survey Department, Plant Pathology Research Institute, ARC, Giza, 12511 Egypt; 6 Department of Plant Production, Faculty of Food & Agricultural Sciences, P.O. Box. 2460, Riyadh, 11451 Saudi Arabia

**Keywords:** Actinobacteria, UPLC–ESI–MS/MS, GC–MS, VOCs, PKSII, NRPS, *phz*E

## Abstract

**Background:**

Actinobacteria from freshwater habitats have been explored less than from other habitats in the search for compounds of pharmaceutical value. This study highlighted the abundance of actinobacteria from freshwater sediments of two rivers and one lake, and the isolates were studied for their ability to produce antimicrobial bioactive compounds.

**Results:**

16S rRNA gene sequencing led to the identification of 84 actinobacterial isolates separated into a common genus (*Streptomyces*) and eight rare genera (*Nocardiopsis*, *Saccharopolyspora*, *Rhodococcus*, *Prauserella*, *Amycolatopsis*, *Promicromonospora*, *Kocuria* and *Micrococcus*). All strains that showed significant inhibition potentials were found against Gram-positive, Gram-negative and yeast pathogens. Further, three biosynthetic genes, polyketide synthases type II (PKS II), nonribosomal peptide synthetases (NRPS) and aminodeoxyisochorismate synthase (*phz*E), were detected in 38, 71 and 29% of the strains, respectively. Six isolates based on their antimicrobial potentials were selected for the detection and quantification of standard antibiotics using ultra performance liquid chromatography (UPLC–ESI–MS/MS) and volatile organic compounds (VOCs) using gas chromatography mass spectrometry (GC/MS). Four antibiotics (fluconazole, trimethoprim, ketoconazole and rifampicin) and 35 VOCs were quantified and determined from the methanolic crude extract of six selected *Streptomyces* strains.

**Conclusion:**

Infectious diseases still remain one of the leading causes of death globally and bacterial infections caused millions of deaths annually. Culturable actinobacteria associated with freshwater lake and river sediments has the prospects for the production of bioactive secondary metabolites.

**Electronic supplementary material:**

The online version of this article (10.1186/s12934-018-0912-0) contains supplementary material, which is available to authorized users.

## Background

Actinobacteria are diverse group of Gram positive and filamentous bacteria that have high guanine–cytosine (GC) content ranging from 50 to 70 mol% in their genome [1]. They are considered excellent elaborators of pharmaceutical products such as antibiotics and industrial enzymes and are well known as a prominent source for finding novel biologically active secondary metabolites [2, 3].

Antibiotic resistance against available drugs is one of the primary reasons to seek new and novel drugs such as antibiotics from a natural source to fight against multidrug-resistant pathogens [4]. The infections caused by globally emerging Gram-negative multidrug-resistant pathogens are an important challenge. Vancomycin-resistant *enterococci* (VRE), Methicillin-resistant *Staphylococcus aureus* (MRSA), extended-spectrum β-lactamase (ESBLs) that produce Gram-negative bacteria, and *Klebsiella pneumoniae* carbapenemase (KPC) that produces Gram-negative bacteria are few of the most significant cases that are gradually increasing in ubiquity and virulence [5]. Due to this fact, there is an incessant requirement for the search for new bioactive compounds from unexplored/less explored environments [6]. As a result, it is important to target such environments that could be highly potent sources of novel and bioactive compounds. Among all living organisms, the actinobacteria phylum currently represents the most prospective group of microorganisms for the discovery of bioactive compounds such as antimicrobials, antitumor agents, antiparasitics, anticancer agents and enzymes [7, 8]. It has been shown that 45% of all reported bioactive compounds of microbial origin are produced by actinobacteria, more than 70% of which are produced by the largest genus in the phylum *Streptomyces* [9].

Since the discovery of the first antibiotic from actinobacteria in 1940, actinomycin, the exploration of these micro-organisms has resulted in the isolation of thousands of naturally occurring antibiotics to date [10]. Several novel species of *Streptomyces* have been reported worldwide as potential natural sources for the discovery of naturally occurring antibiotics [11–13]. Actinobacteria have been extensively reported from different ecosystems such as soil, freshwater, marine and as endophytes from plants [14, 15] and have been investigated for their potential contributions to the pharmaceutical industry by different researchers [16–22]. However, there has been a significant decline in the rate of discovery of novel actinobacteria in recent years [23, 24]. Therefore, the exploration of potential actinobacteria from unexplored habitats is an important approach to discovering novel antibiotics to meet the current needs [25, 26].

Northeast India is a large bioprospecting area that was identified as the Indo-Burma mega-biodiversity hotspot by Conservation International, and the area is well known for its rich biodiversity and unexplored biological resources [27, 28]. Bioprospection studies on the actinobacteria phylum have mainly focused on terrestrial and marine ecosystems, and few have focused on freshwater ecosystems [29]. There are several reports that have examined the diversity of actinobacteria in freshwater worldwide [29–31], but very few studies have reported on their biosynthetic potential. Therefore, it will be of great importance to characterize the various biologically active secondary metabolites produced by actinobacteria obtained from freshwater sediments. The present study intended to isolate actinobacterial cultures, screen them for in vitro antimicrobial inhibitory activity, detect their bioactive secondary metabolites and phylogenetically identify the potential antibiotic-producing actinobacteria from freshwater sediments of selected freshwater lakes and rivers in India.

## Methods

### Sediment sampling

Samples were collected from two rivers [Tlawng River (24°52′N; 92°36′E), Tuirial River (24°21′N 92°53′)] and one lake [Tamdil Lake (23°44′N; 92°57′E)] (Additional file 1: Fig. S8). Samples were randomly collected from five different stations of each river and lake at average depths of 2–5 m. The labeled samples were placed in sterile tubes (50 ml), transported to the laboratory and were processed immediately for the isolation of actinobacteria.

### Isolation of freshwater actinobacterial strains

The collected samples were subjected to physical pretreatment (55 °C for 6 min) to hinder the growth of fast-growing bacteria and favor the growth of actinobacteria [7]. Actinobacteria were isolated using the serial dilution method and the spread plate technique. The stock solution of the sample was prepared with 1 ml of water sediment (water + sediment suspension) and 9 ml of sterile distilled water in a test tube, and the solution was mixed for 10 min. The suspension was serially diluted by transferring 1 ml aliquots to a series of test tubes; each containing 9 ml of sterile distilled water to prepare the final volumes of 10^−1^, 10^−2^ and 10^−3^, and the diluted suspension was spread over the surface of selected nutritional media. Seven selective media, starch casein agar (SCA), yeast extract-malt extract agar (ISP2), Actinomycetes isolation agar (AIA), Streptomyces agar (SA), glycerol–asparagine agar (ISP5), tyrosine agar medium (ISP7), and tap water yeast extract agar (TWYE), were supplemented with nalidixic acid (30 mg/ml) and cyclohexamide (30 mg/ml) to inhibit the growth of Gram-negative bacteria and fungi, respectively. The plates were incubated at 28 ± 1 °C for 7–30 days, and the colonies were observed periodically. Pure cultures were obtained after two to three successive sub-culturing rounds and transferred to fresh isolation media. The cultures were preserved in their respective slants at 4 °C and 30% glycerol at − 80 °C.

### Morphological and microscopic characterization of actinobacterial strains

Pure cultures of the isolates were identified based on their morphological and cultural characteristics following the International *Streptomyces* Project (ISP) [32]; the nature of the colony, the color of aerial and substrate mycelium, the production of diffusible pigments and the utilization of carbon source were studied [33]. The spore chain morphologies of the isolates were studied using a scanning electron microscope (SEM). The mycelium structures were observed using a phase contrast microscope (Olympus), and the organisms were identified according to *Bergey’s Manual of Determinative Bacteriology* 9th edition.

### Molecular identification and phylogenetic analysis

Genomic DNA was isolated and purified using a DNA extraction kit (Invitrogen) as described in previous studies [21]. Ribosomal RNA (16S rRNA) genes were amplified using universal bacterial primers [34]. The reactions and conditions of the PCR were performed exactly as reported in our previous studies [21], and sequencing was done commercially at Sci Genome Pvt. Ltd. Cochin, India. Sequences were compared with the reference strains of actinobacteria from the NCBI genomic database using a BLASTn search to determine similarity percentages. The strains with highest similarity percentages were retrieved from the EzTaxon database [35], and multiple sequence alignment was performed using Clustal W software packaged in MEGA 6.0 [36]. The evolutionary models were selected based on the lowest Bayesian information criterion (BIC) scores and the highest Akaike information criterion (AIC) values using MEGA 6.0 [37]. Phylogenetic analysis was performed using MEGA 6 software using the maximum-likelihood method and using the Tamura Nei parameters algorithm taking *E. coli* as the outgroup [38]. The significance of the branching order was determined by bootstrap analysis of 1000 alternative trees. The obtained nucleotide sequences of the 16S rRNA gene fragments were deposited, and accession numbers were acquired. Trees were viewed and edited using the FigTree 1.3.1 program.

### Screening for antimicrobial activity

The antimicrobial activities of the actinobacterial isolates were tested against five bacterial pathogens [Gram-positive bacteria: *Staphylococcus aureus* MTCC-96, *Bacillus subtilis* NCIM-2097, and *Micrococcus luteus* NCIM-2170; Gram-negative bacteria: *Pseudomonas aeruginosa* MTCC-2453 and *Escherichia coli* MTCC-739 and yeast: *Candida albicans* MTCC-3017]. The pathogens were obtained from the Microbial Type Culture Collection (MTCC), Chandigarh and National Collection of Industrial Microorganisms (NCIM), Pune, India. The crude extracts were prepared by inoculating a single purified colony of actinobacteria in Tryptone yeast extract broth medium (ISP medium 1) and incubated at 28 °C, 150 rpm for 7–20 days. The grown cultures after centrifugation were used to assess antimicrobial activity by the agar well diffusion method [39]. The test pathogenic bacteria were spread on a nutrient agar plate, 6 mm diameter wells were prepared using a sterile cork borer, 70 µl of the clear supernatant of the actinobacteria was dispensed into individual wells, and the plates were incubated at 28 ± 2 °C for 24 h. The anti-microbial activity of the isolates was evaluated as described by Zothanpuia et al. [21].

### Antimicrobial assay using crude extract

The actinobacteria isolates that were selected based on antimicrobial screening were grown in ISP1 broth using a 500 ml conical flask at 28 °C in a shaker incubator for 30 days. The filtrates of the grown cultures were used for the extraction using methanol 1:1 ratio (v/v). The methanolic crude extracts of the isolates were prepared in concentrations of 1, 2, 5, 20 mg and 40 mg/ml [21] with sterile water and used for antimicrobial activity by the agar well diffusion method and disk diffusion assay [39, 40].

### Determination of MIC

The minimum inhibitory concentration (MIC) of the selected strains was determined using the broth micro dilution technique in a 96-well microtiter plate [41]. The methanolic extracts of the strains were dissolved and diluted in different concentrations (0.025, 0.05, 0.1, 0.2, 0.4, 0.8, 1.6 and 3.2 mg/ml) and were used to test the antimicrobial activity by growing them with bacterial culture in a 96-well microtiter plate. The ampicillin (1 mg/ml) amended bacterial culture was used as the positive control, and the bacterial cultures without treatment were used as the negative control. The plates were incubated at 37 °C for 36 h, and absorbance was taken at 700 nm in a UV–VIS spectrophotometer (MultiscanTM GO, Thermo Scientific, MA, USA). EC50 was expressed and calculated as previously described [21].

### Amplifications of biosynthetic genes (PKS, *phz*E and NRPS)

The presence of biosynthetic genes [Polyketide synthase type II (PKS II) non-ribosomal peptide synthetase (NRPS) and aminodeoxyisochorismate synthase (*phz*E)] was evaluated using degenerate primers for highly conserved regions encoding enzymes associated with the biosynthesis of polyketides, peptides and phenazine, respectively. The primers that were employed and the PCR conditions for the amplification of PKS-II, *phz*E and NRPS gene fragments were described in previous studies [7, 21].

### Phylogenetic analysis PKS II, NRPS and *phz*E gene

Biosynthetic gene sequences of PKS type II, NRPS and *phz*E from the selected seven strains were compared with the sequences from NCBI database using the BLASTn search tool [38] and were aligned by Clustal W software packaged in MEGA 6.0 [36]. The evolutionary model for PKS II, NRPS and *phz*E gene was selected based on lowest BIC value and highest AIC value using MEGA 6.0 [39]. Phylogenetic tree was constructed by the maximum likelihood method using MEGA 6.0 software with General Time Reversible (GTR + G) model for PKS II and Tamura 3-parameter (T92 + G) for NRPS and *phz*E gene [38, 39].

### Detection of antibiotics using UPLC–ESI–MS/MS

Ultra-performance liquid chromatography (UPLC–ESI–MS/MS) was employed to detect antibiotics in the methanolic extracts of the selected strains. Four antibiotics (trimethoprim, fluconazole, ketoconazole and rifampicin) were selected, and a standard solution was prepared using methanol (Additional file 1: Table S4). The mixed standards were diluted in the ranges from 0.5 to 500 ng/ml, and a standard calibration curve was prepared. Instrumentation and analytical conditions were performed using the standardized methods as described in our previous paper (Fig. 4) [21].

### GC–MS analysis

Gas chromatography–mass spectroscopy (GC–MS) was used to determine the volatile organic compounds (VOCs) present in the methanolic extracts of the selected strains. For GC–MS, the Clarus 680 GC was used in the analysis employed with a fused silica column packed with Elite-5MS (5% biphenyl 95% dimethylpolysiloxane, 30 m × 0.25 mm ID × 250 μm *df*), and the components were separated using helium as carrier gas at a constant flow of 1 ml/min. The injector temperature was set to 260 °C during the chromatographic run. A total of 1 μl of the extracted sample was injected into the instrument, and the oven temperatures were as follows: 60 °C (2 min); followed by 300 °C at a rate of 10 °C/min; and 300 °C for 6 min. The mass detector conditions were a transfer line temperature of 240 °C; an ion source temperature of 240 °C, an ionization mode electron impact at 70 eV, a scan time of 0.2 s and a scan interval of 0.1 s. The fragments from 40 to 600 Da were analyzed. The spectra of the detected compounds were compared with their mass spectra from the database of known components stored in the GC–MS NIST (2008) library.

### Statistical analysis

All experiments were conducted in triplicate, and the readings were taken as the mean ± the standard deviation of the mean of three replicates, which were calculated using Microsoft Excel XP 2010. One-way analysis of variance (ANOVA) was performed to analyzed significant difference (P = 0.05) between antimicrobial activities obtained isolates by using SPSS software version 20.0.

## Results

### Isolation and distribution of freshwater actinobacteria

A total of 68 isolates of actinobacteria were obtained from freshwater sediments; 30 strains from Tamdil Lake, 19 from Tlawng River, 19 from Tuirial River. From a total of seven different media employed for isolation, 31 isolates were recovered from the SCA medium, 24 from AIA, 3 from ISP5, 4 from SA, 1 from TWYE, 3 from ISP7, and 2 from ISP2. These results clearly indicated that SCA was the most suitable medium for the isolation of actinobacteria from freshwater sediments and yielded 45% of the total isolates followed by AIA (36%). After 30 days of incubation, the cultures matured and the actinobacteria colony was observed to exhibit white, black, yellow, orange, brownish white and pale yellow colors. Most of the actinobacterial strains analyzed by field emission gun-scanning electron microscopy (FEG-SEM) showed that the aerial mycelia produce spiral spore chains (Additional file 1: Fig. S1).

### Molecular characterization and phylogenetic affiliation

**Fig. 1:**
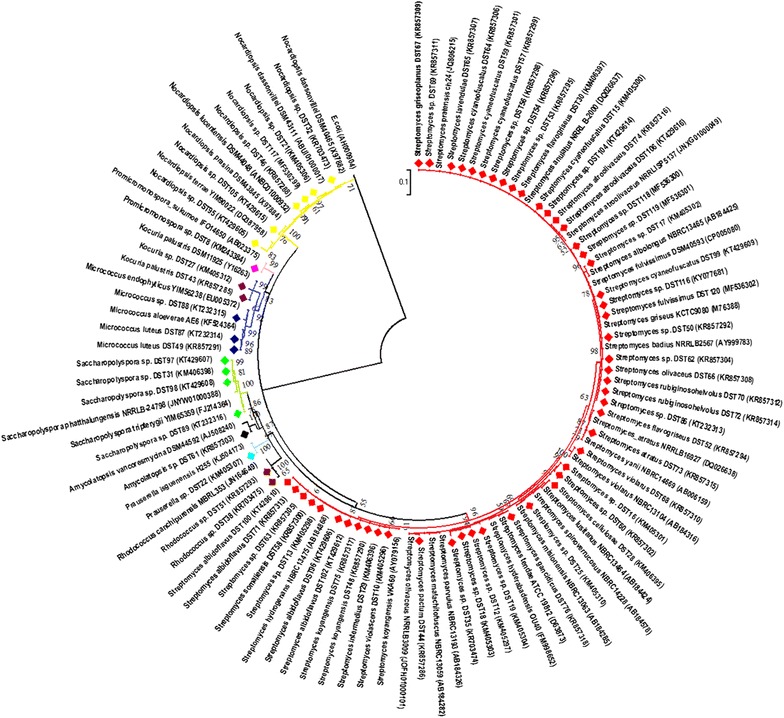
Maximum likelihood phylogenetic tree constructed using Tamura-Nei model based on 16S rRNA gene sequences of actinobacteria showing the phylogenetic relationship between the isolates with closest type strain sequences. Numbers at branches indicate bootstrap values in 1000 replicates

According to the molecular identification using 16S rRNA gene sequencing, 68 actinobacteria isolates were classified into six families and nine genera with similarity percentages ranging from 98 to 100%. The majority of the isolates were grouped under *Streptomycetaceae* followed by *Pseudonocardiaceae, Nocardiopsaceae*, *Nocardiaceae, Promicromonosporaceae* and *Micrococcaceae*. *Streptomyces* was the most dominant genus (n = 49, 72%), and the other genera included *Nocardiopsis* (n = 6), *Saccharopolyspora* (n = 4), *Rhodococcus* (n = 2), *Prauserella* (n = 1), *Amycolatopsis* (n = 1), *Promicromonospora* (n = 1) *Kocuria* (n = 1) and *Micrococcus* (n = 3) (Additional file 1: Table S1). All sequences were deposited in the NCBI GenBank database, and accession numbers were given (KM243384, KM405296–KM405298, KM405300–KM405304, KM405306–KM405307, KM405310, KM406395, KM406397, KM406398, KR703473–KR703475, KR857285, KR857286, KR857288, KR857290–KR857296, KR857298–KR857318, KT232313–KT232316, KT429605–KT429610, KT429612, KT429614–KT429616, KY077681, MF536299–MF536302). The length of the sequences was used for the construction of phylogenetic tree ranges from 500 to 1000 bp. The phylogenetic tree was constructed using the maximum-likelihood and Tamura Nei parameters with the lowest BIC values (12,154.636) and highest AIC values (10,215.261) (Fig. 1). The topology of the tree that was generated differentiated the isolates into 3 major clades. All genera of *Streptomyces* formed major clade I with a bootstrap support value of 96%. Rare genera, such as *Saccharopolyspora, Amycolatopsis* and *Prauserella*, which fell under the family *Pseudonocardiaceae*, were clustered together with *Rhodococcus* and had a bootstrap value of 87%. *Micrococcus* and *Kocuria* under the family *Micrococcaceae* formed a separate clade II with *Promicromonospora* and had a bootstrap value of 83%. All strain types of *Nocardiopsis* formed a separate clade III with a bootstrap value of 100%.

### Relative abundance

**Fig. 2:**
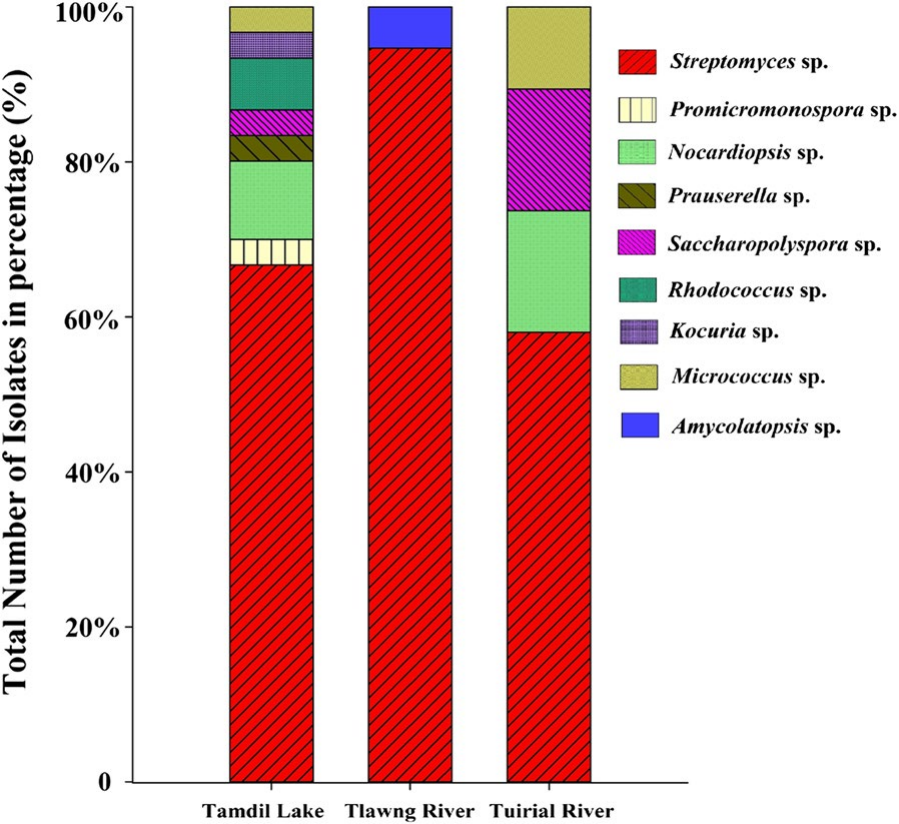
Abundance of actinobacterial isolates in three fresh water systems

The relative abundance of actinobacteria at the genus level revealed that *Streptomyces* was the most dominant in Tamdil Lake (n = 20, 40.8%) followed by Tlawng River (n = 18, 36.7%) and Tuirial River (n = 11, 22.4%) from a total of 49 isolates. However, some rare actinobacteria, such as *Promicromonospora* sp., *Prauserella* sp., *Rhodococcus* sp., and *Kocuria* sp., were obtained from only Tamdil Lake, while *Amycolatopsis* sp. was found in only Tlawng River. *Saccharopolyspora* sp., *Nocardiopsis* sp. and *Micrococcus* sp. were obtained from Tamdil Lake and Tlawng River, whereas several different species of *Streptomyces* were obtained from all study sites (Fig. 2). These results showed that the freshwater actinobacteria population varies substantially between lakes and rivers. In comparison to the river ecosystem, the lake ecosystem was observed to be more favorable for actinobacterial growth, as indicated by the enhanced number of isolates obtained with greater diversity.

### Evaluation of antimicrobial activity

Initially, all isolates (n = 68) were subjected to preliminary screening against five bacterial pathogens (*S. aureus*, *B. subtilis, M. luteus*, *P. aeruginosa, E. coli*) and yeast (*C. albicans*). All isolates exhibited antagonistic activity against at least three of the six tested pathogens. *E. coli* was found to be the most susceptible pathogen and all isolates (100%) showed activity against it within the inhibition range of 7.4 mm to 15.5 mm diameter (Additional file 1: Table S2). This was followed by *P. aeruginosa* (95.65%), *C. albicans* (79.71%), *B. subtilis* (78.26%) and *S. aureus* (63.76%). Only 26 (37%) isolates showed positive activity against *M. luteus*. The maximum activity was recorded by *Streptomyces flavogriseus* strain DST30 (18.8 mm) followed by *Streptomyces cyaneofuscatus* strain DST57 (15.95 mm) and *Streptomyces albidoflavus* DST71 (15.9 mm) against *M. luteus, C. albicans and B. subtilis*, respectively. Six strains (*Streptomyces* sp. DST25, *Streptomyces cellulosae* DST28*, Streptomyces flavogriseus* DST52, *Streptomyces albidoflavus* DST71, *Streptomyces* sp. DST116 and *Streptomyces* sp. DST119) showed broad-spectrum antimicrobial activities (Table 1), inhibiting all six tested pathogens, and these strains were selected as potential candidates for further investigation. Mean (± SD) followed by the same letter(s) in each column are not significantly different at P < 0.05 using Duncan’s new multiple range test.

**Table 1:** Antimicrobial activity of selected strains of actinobacteria

Strain	Antibacterial properties					Yeast	Biosynthetic genes		
	*E. coli*	*P. aeruginosa*	*S. aureus*	*M. luteus*	*B subtilis*	*C. albicans*	PKS-II	NRPS	*phz*E
*Streptomyces* sp. DST25	9.40 ± 0.03^a^	12.0 ± 0.06^a^	9.00 ± 0.06^a^	13.2 ± 0.10^a^	12.5 ± 0.2^a^	12.5 ± 0.20^a^	+	+	−
*Streptomyces cellulosae* DST28	9.50 ± 0.01^a^	10.0 ± 0.10^bc^	10.0 ± 0.10^bc^	12.7 ± 0.10^a^	12.5 ± 0.1^a^	11.5 ± 0.50^bc^	−	−	−
*Streptomyces flavogriseus* DST52	15.0 ± 0.01^bc^	11.5 ± 0.15^a^	8.00 ± 0.05^bde^	10.8 ± 0.10^bc^	8.4 ± 0.00^bc^	15.5 ± 0.05^bde^	+	+	−
*Streptomyces albidoflavus* DST71	13.0 ± 0.30^bde^	10.0 ± 0.04^bc^	6.60 ± 0.40^bdf^	6.20 ± 0.20^bde^	15.9 ± 0.2^bde^	13.8 ± 0.10^bdfg^	−	+	+
*Streptomyces* sp. DST116	9.00 ± 0.30^a^	8.50 ± 0.05^bde^	9.20 ± 0.25^a^	18.8 ± 0.10^bdfg^	14.4 ± 0.2^bdfg^	12.9 ± 0.05^a^	+	+	−
*Streptomyces* sp. DST119	8.10 ± 0.10^bdf^	7.00 ± 0.10^bdf^	8.00 ± 0.10^bde^	14.3 ± 0.10^bdfh^	14.2 ± 0.1^bdfg^	13.1 ± 0.10^bdfh^	+	+	+

Mean (± SD) followed by the same letter(s) in each column are not significant different at P < 0.05 using Duncan’s new multiple range test

### Antimicrobial activity using methanol crude extract

The methanolic crude extracts of the six selected strains that were tested for their antimicrobial activity showed adequate inhibition zones at 20 and 40 mg/ml (Additional file 1: Fig. S1) for all six samples, while all the isolates showed no activity in 1 and 2 mg/ml. The agar well diffusion assay showed better results compared to the filter paper disk diffusion assay.

### MIC of selected strains

The methanolic crude extracts of the six strains were subjected to antimicrobial activity quantification by determining the MIC of each strain against six pathogens. *Streptomyces* sp. DST116 showed maximum activity against *M. luteus* (EC50 = 0.05103 mg/ml) among all tested pathogens. *Streptomyces cellulosae* DST28 (EC50 = 0.3371 mg/ml) and *Streptomyces flavogriseus* DST52 (EC50 = 0.003 mg/ml) also showed the highest antimicrobial activities against *M. luteus*. *Streptomyces* sp. DST25 showed the highest activity against *B. subtilis* (EC50 = 0.009 mg/ml), and *Streptomyces albidoflavus* DST71 showed the highest activity against *P. aeruginosa* (EC50 = 0.05042 mg/ml) (Table 2).

**Table 2:** EC_50_ of six *Streptomyces* strains against six pathogens

Strain	EC50 mg/ml					
	*E. coli*	*P. aeuginosa*	*S. aureus*	*B. subtilis*	*M. luteus*	*C. albicans*
*Streptomyces* sp. DST116	0.235	0.231	0.110	0.227	0.051	0.069
*Streptomyces cellulosae* DST28	1.673	2.353	1.085	0.804	0.331	0.900
*Streptomyces* sp. DST25	0.086	0.144	0.070	0.009	0.286	0.070
*Streptomyces* sp. DST119	0.260	0.015	0.015	0.278	0.950	1.195
*Streptomyces flavogriseus* DST52	0.056	0.267	0.040	0.170	0.003	1.600
*Streptomyces albidoflavus* DST71	0.102	0.050	0.138	0.650	0.190	0.075

### Biosynthetic gene analysis

Out of the 68 isolates screened for a biosynthetic gene, NRPS was detected in 71% (n = 49) of the isolates, PKS type II was detected in 26 isolates (38%), and *phz*E was detected in 28% (n = 19) of the isolates (Additional file 1: Table S2). A total of 11 isolates (DST45, DST47, DST54, DST56, DST57, DST58, DST74, DST76, DST77, DST99, DST101) were found to have all three genes.

### Phylogenetic analysis of biosynthetic genes

**Fig. 3:**
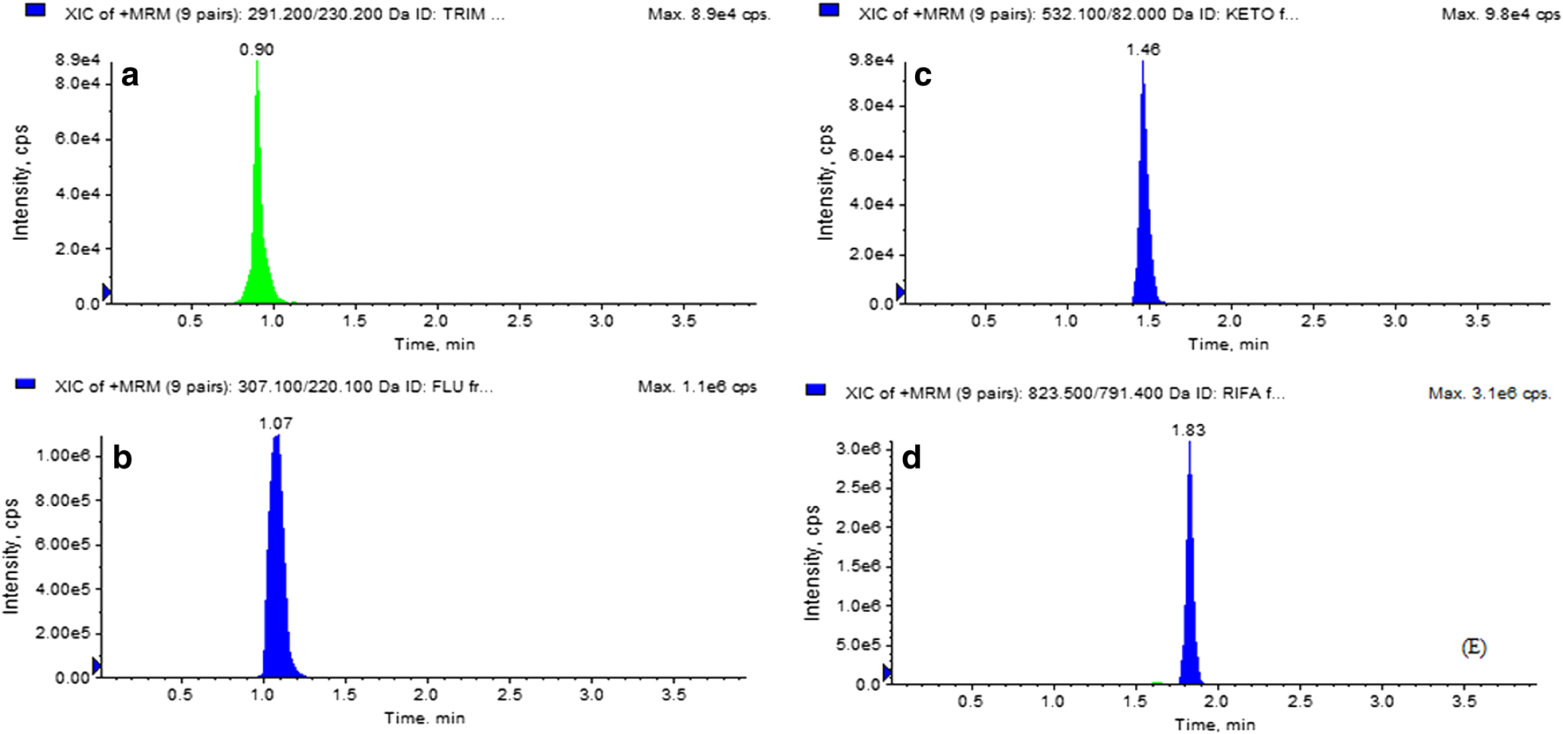
Maximum likelihood (ML) phylogenetic tree constructed using amino acid sequences for **a** PKS type II gene; **b** NRPS gene and **c** phzE gene. The scale bar represents the amino acid changes

The nucleotide sequences of three biosynthetic genes (PKS II, NRPS and *phz*E) showed 82–92% similarity with the type strain from NCBI-BLASTn database. The transition and transversion bias ratio of PKSII, NRPS and *phz*E gene was 0.55, 0.33 and 0.17 respectively whereas the maximum log likelihood for the substitution computation was − 2765.453, − 501.484 and − 801.607 respectively. The phylogenetic tree constructed using PKS II sequences revealed that *Streptomyces* sp. DST29 formed separate clade with *Streptomyces* sp. MM48 *Streptomyces gobitricini* with bootstrap values of 99% while *Streptomyces* sp. DST116, *Streptomyces* sp. DST52 and *Streptomyces* sp. DST119 each formed a separate clade with a bootstrap support value of 99–100% (Fig. 3a). Similarly the NRPS gene sequences of *Streptomyces* sp. DST116 *Streptomyces* sp. DST25, *Streptomyces* sp. DST71 and *Streptomyces* sp. DST119 formed separate clade with *Streptomyces* sp. CAH29-18, *Streptomyces albidus* NBRC14052, *Streptomyces cyaneofuscatus* DST103, *Streptomyces bamensis* NBRC14727 and *Streptomyces* sp. BSH50-42 respectively with a bootstrap value of 84–89% (Fig. 3b). Similarly *Streptomyces* sp. DST119 and *Streptomyces* sp. DST71 were clustered separately in *phz*E gene sequences forming same clade with *Streptomyces* sp. HB291 and *Streptomyces* sp. 13–33–9 respectively with a bootstrap value of 100% (Fig. 3c).

### GC–MS analysis

The methanolic crude extracts of the six selected strains were investigated to determine their volatile organic compounds using GC–MS, which revealed thirty-five VOCs (Additional file 1: Table S3). Fourteen compounds were detected from the extract of *Streptomyces albidoflavus* DST71 within the retention time of 15–29 min (Additional file 1: Fig. S2). Among the compounds, hexanal constituted the maximum amount, which accounted for 23.2% of the total volume. Six VOCs, valine, glutaraldehyde, d-leucine, 3,3-dimethyl-4-methylamino-butan-2-one, pentadecylamine, cyclopropane and 1-butyl-2-(2-methylpropyl)-, were detected from the extract of *Streptomyces* sp. DST25, and glutaraldehyde was the most abundant followed by an amino acid, valine (Additional file 1: Fig. S3). Only one compound (di-*n*-octyl phthalate) was detected in extracts of *Streptomyces cellulosae* DST28 (Additional file 1: Fig. S4). Seven compounds were determined from the extract of *Streptomyces flavogriseus* DST52, of which carbonic acid, 2, 2, 2-trichloroethyl undec-10-enyl ester alone constituted 49.78% (Additional file 1: Fig. S5). Only 2-methoxy-4,5-diphenyl-6-(2′-phenylethyl)pyrimidine was detected in the extract of *Streptomyces* sp. DST116 (Additional file 1: Fig. S6), while six compounds were detected in the extract of *Streptomyces* sp. DST119 in which 2-benzylthio-8-methyl-7-phenylpyrano [2,3-f]benzoxazol-6(h)-one constituted the maximum amount (42.66%) (Additional file 1: Fig. S7).

### Detection and quantification of antibiotics using the UPLC-MRM method

**Fig. 4:**
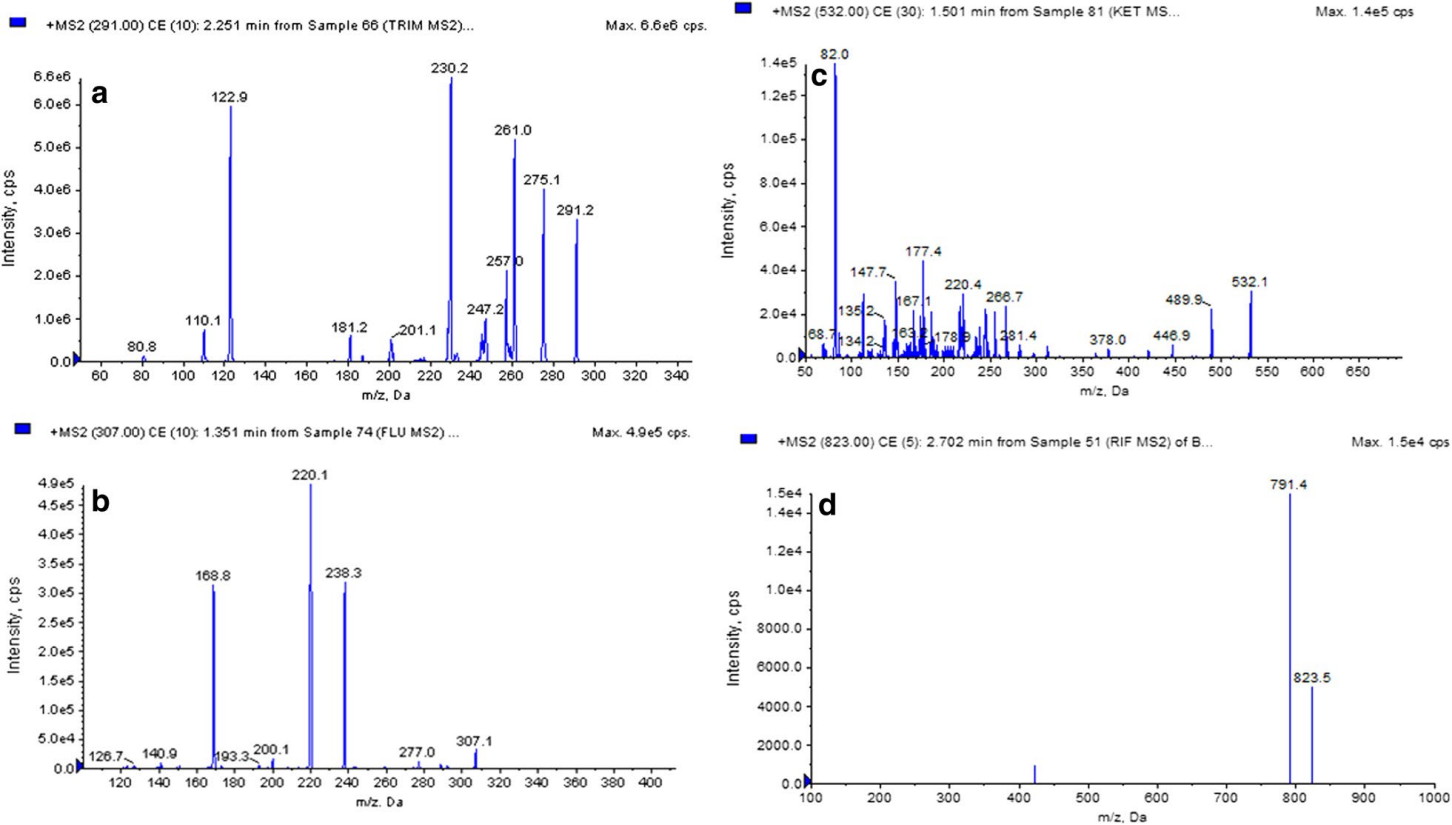
MRM extracted ion chromatogram of reference analyte: **a** trimethoprim, **b** fluconazole, **c** ketoconazole, **d** rifampicin

**Fig. 5:**
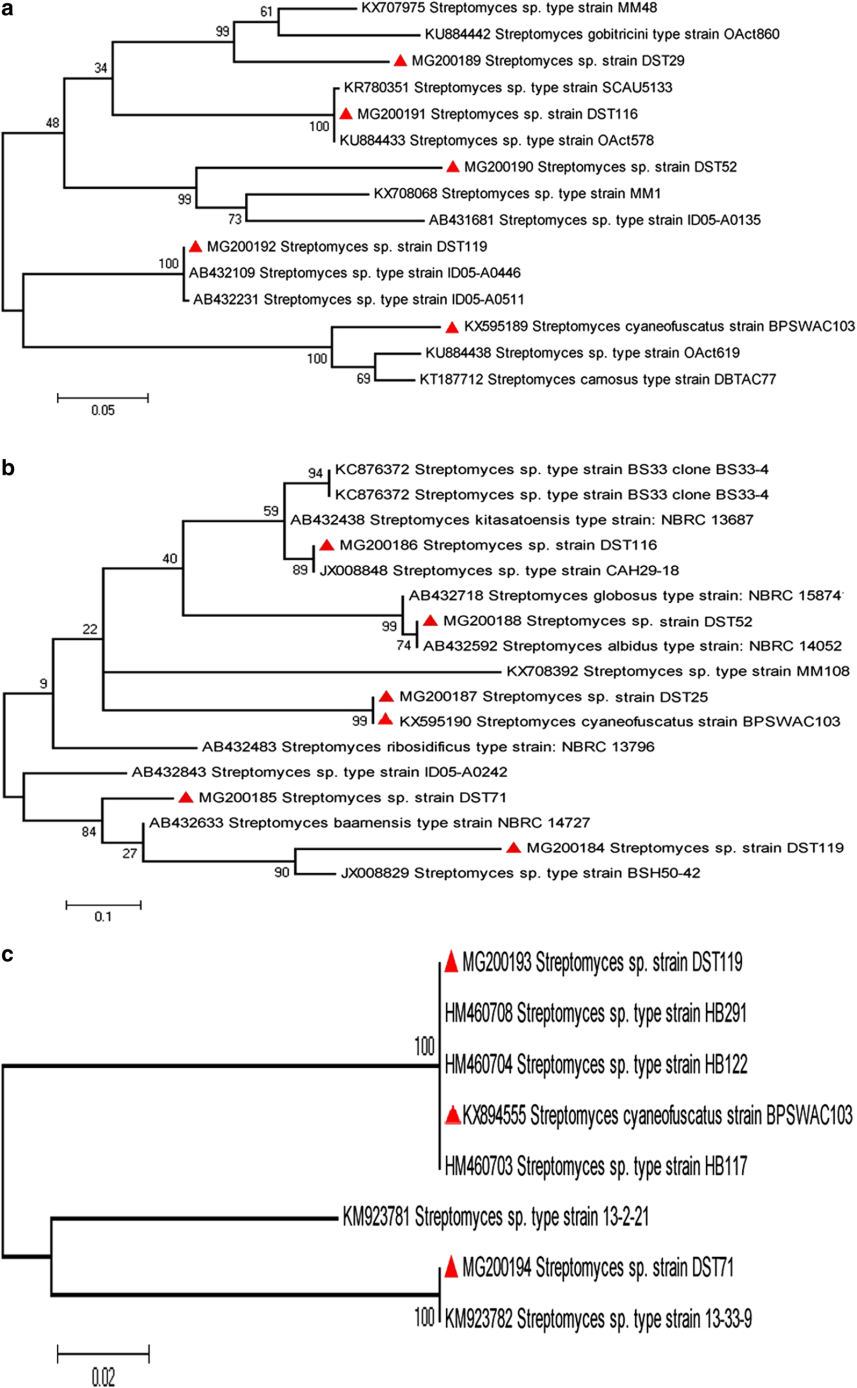
MS/MS Spectra of reference analytes; **a** trimethoprim, **b** fluconazole, **c** ketoconazole, **d** rifampicin (as per [21])

The UPLC–ESI–MS/MS analysis for detection of certain standard antibiotics of the methanolic crude extracts of the selected isolates showed that rifamycin was present in the highest amount in all samples followed by ketoconazole, trimethoprim and fluconazole. Trimethoprim was found to be present in higher amounts (39 μg/g) in extracts of *Streptomyces flavogriseus* DST52 compared to the other samples. Extracts of *Streptomyces cellulosae* DST28 contained more fluconazole (17 μg/g), ketoconazole (50 μg/g) and rifamycin in (74 μg/g) compared to other samples (Table 3 and Figs. 4, 5). MS/MS Spectra of standard reference analytes i.e. trimethoprim, fluconazole, ketoconazole and rifampicin showed as Fig. 5 was used from our earlier publication [21].

**Table 3:** Antibiotics content of six selected strains (μg/g)

Strain no.	Trimethoprim	Fluconazole	Ketoconazole	Rifamycin
*Streptomyces* sp. DST25	17.0	8.0	29.0	51.0
*Streptomyces cellulosae* DST28	21.0	17.0	50.0	74.0
*Streptomyces flavogriseus* DST52	39.0	5.0	28.0	78.0
*Streptomyces albidoflavus* DST71	27.0	16.0	35.0	68.0
*Streptomyces* sp. DST116	26.0	10.0	49.0	86.0
*Streptomyces* sp. DST119	28.0	6.0	32.0	64.0

## Discussion

The bio-resources in freshwater ecosystems are largely unexplored, especially in the field of microbiology. Freshwater ecosystems are becoming a promising area for the isolation of bioactive compounds of pharmaceutical and biotechnological importance [21]. In the present investigation, 68 actinobacterial strains were isolated from three freshwater systems, and maximum strains were obtained from the lake sediment compared to the sediments from the two rivers. This could be because sediments containing actinobacteria in rivers are continuously removed by running water and get deposited in different areas throughout the river. At the same time, lake sediments are concentrated in particular areas that are not drastically affected by running water. Different nutritional media were employed to achieve maximum diversities of actinobacteria, since nutrient uptake differed between organisms. The results indicated that SCA was the best medium for the isolation of the maximum number of actinobacteria strains, which was in accordance with earlier studies [42–45].

*Streptomyces* represents the largest genus under the bacteria domain [46] and the actinobacteria phylum [21]. The present investigation also showed that *Streptomyces* was the most dominant genus in freshwater sediments, which is in accordance with the findings of Wohl and McArthur. [47]; Deshmukh and Sridhar. [42]; Ningthoujam et al. [48]; Sanasam et al. [49]; Jami et al. [50] and Zothanpuia et al. [45]. There are also several genera of actinobacteria other than *Streptomyces* that are called rare genera whose isolation frequencies were lower compared to *Streptomyces* [51]. Only 12% of the actinobacterial isolates that were recovered were rare genera, which included *Kocuria*, *Nocardiopsis*, *Amycolatopsis*, *Saccharopolyspora*, *Rhodococcus*, *Prauserella*, *Promicromonospora* and *Micrococcus*, and these genera have been previously reported from freshwater habitats [29, 49, 50, 52]. To the best of our knowledge, *Amycolatopsis, Prauserella* and *Promicromonospora* have not yet been reported from freshwater sediments and were isolated for the first time in the present study. However, halophilic actinobacteria, *Amycolatopsis halophila* [53], *Prauserella salsuginis, Prauserella flava, Prauserella aidingensis,* and *Prauserella sediminis* [54], were reported to be isolated from a saline lake in Xinjiang Province, northwest China, while *Promicromonospora thailandica* has also been reported from marine sediment [55].

Actinobacteria are a potential candidate to fight against multidrug-resistant organisms and are well-known producers of antimicrobial compounds, and actinobacteria have been found in different habitats worldwide [4, 20, 21, 40]. The present study reports the antimicrobial activity of sixty-eight actinobacterial isolates that showed activity against at least three of the tested pathogens. Some rare genera of actinobacteria, such as *Kocuria*, *Nocardiopsis*, *Amycolatopsis*, *Saccharopolyspora*, *Rhodococcus*, *Prauserella*, *Promicromonospora* and *Micrococcus*, were also evaluated for their antimicrobial activities in the present study. Among them, *Saccharopolyspora* sp. DST31, *Nocardiopsis* DST32, *Rhodococcus* sp. DST38 and *Nocardiopsis* DST95 showed activity against five of the six tested pathogens. Sibanda et al. [29] isolated actinobacteria belonging to *Saccharopolyspora* and *Actinosynnema* from the Tyume River, South Africa, and found antibacterial activity against the tested pathogens, which supports the present investigation. Several other rare genera of actinobacteria from freshwater habitats, except for *Amycolatopsis, Prauserella* and *Promicromonospora*, have been previously reported for their antimicrobial activity, as discussed earlier.

Six potential *Streptomyces* strains that showed a broad spectrum of antimicrobial activities against all tested pathogens were further selected, and the methanolic extracts of the strains showed better activity using the agar well diffusion method compared to the filter paper disk diffusion assay, which was supported by the findings of Gebreyohannes et al. [40]. Recently, we recorded the potential microbial activity of *Streptomyces cyaneofuscatus* from freshwater sediments of Tamdil Lake [21]. Broad-spectrum antimicrobial activity was also measured in *Streptomyces* sp. AZ-NIOFD1 from the Nile River [56]. Various potential strains of *Streptomyces* have also been identified in freshwater habitats [21, 57–59]. The methanolic crude extract of *Streptomyces flavogriseus* DST52 showed antimicrobial activity with an MIC value of 0.003 mg/ml, which was lower than the actinobacterial strain SMS_SU21 from a mangrove ecosystem that showed antimicrobial activity with an MIC value of 0.05 mg/ml [60]. The MIC of *Streptomyces* sp. DST119 extract was 0.015 mg/ml against *S. aureus*, while that of *Streptomyces flavogriseus* DST52 was 0.056 mg/ml, which was far lower than those of the actinobacterial crude extract (1.65 and 1.84 mg/ml) against *S. aureus* and *E. coli*, respectively [40].

PKS-II, *phz*E and NRPS have been extensively described as responsible for the synthesis of a broad range of structurally diverse secondary metabolites in actinobacteria [7, 21, 61]. PKS and NRPS are responsible for the synthesis of bioactive polyketides and peptides, while phenazine is an antibiotic that has been reported to be derived from *phz*E, and all three genes are all renowned for playing vital roles in biological control [7, 21, 62]. The present study also correlated antimicrobial compounds with reference to their biosynthetic genes in some of the selected strains. Among the selected strains that showed antimicrobial activity against all tested pathogens, the biosynthetic genes PKS-II, *phz*E and NRPS were all detected and amplified with the expected size in *Streptomyces* sp. DST116 and DST119. However, none of the genes were detected in *Streptomyces cellulosae* DST28, which clearly showed that the strains that show antimicrobial activity do not necessarily contain PKS-II, *phz*E or NRPS genes, and these findings are in agreement with previous studies [63, 64]. The biosynthetic genes for six selected *Streptomyces* strains were sequenced and deposited in NCBI database and Genbank accession number were given as MG200184–MG200188 for NRPS; MG200189–MG200192 for PKSII; MG200193–MG200194 for *phz*E.

In the present study, four antibiotics were detected and quantified using the UPLC–ESI–MS/MS method. This method has been successfully employed to quantify bioactive compounds such as antibiotics and phenolic compounds [15, 21]. Antibiotics such as rifamycin and trimethoprim were detected and quantified from the crude methanol extract of six *Streptomyces* strains, which was supported by the findings of Passari et al. [19]. Fluconazole and ketoconazole were also quantified in all selected extracts of *Streptomyces* strains, which were also recently reported from *Streptomyces cyaneofuscatus* isolated from Tamdil Lake, Northeast India [21].

Secondary metabolite profiling based on GC–MS is becoming a foundation in the field of biological sciences and has been successfully employed to determine VOCs from various samples [20, 21]. The actinobacteria phylum has been reported as prolific producers of thousands of bioactive secondary metabolites. The present investigations measured 35 VOCs from six methanolic extracts of *Streptomyces* strains, of which maximum compounds were retrieved from *Streptomyces albidoflavus* DST71. Among the identified compounds from extracts of *Streptomyces albidoflavus* DST71, all except oxirane, 2-butyl-3-methyl-, cis, azacyclodecan-5-ol, *N*-(4-chlorobenzenesulfonyl)azetidin-3-one and 1,3,5-triazaadamantane detected compounds that have the antimicrobial activity, as reported by earlier researchers [65–73]. In the present study, the amount of hexanal in the methanol extract of *Streptomyces albidoflavus* DST71 was found to be maximum (23.2%), and this compound was reported to be one of the constituents of the crude extract of the roots of *Leonurus sibiricus* for its antibacterial, anti-inflammatory, antioxidant, and antiproliferative properties [70]. Antimicrobial activities of 2-thiophenecarboxylic acid, 5-(1, 1-dimethylethoxy)- and heptanal have also been observed in the extracts of *Phormidium autumnale* and *Chlorella vulgaris*, respectively [69]. The antimicrobial activity of glutaraldehyde was also discussed earlier [66, 74] and was also measured in the extract of *Streptomyces* sp. DST25. All compounds extracted from the crude extract of *Streptomyces* sp. DST25 except cyclopropane, 1-butyl-2-(2-methylpropyl)-were previously reported in antimicrobial studies [71, 75, 76]. The amino acid valine was also determined as a major compound next to glutaraldehyde in the present study, and this compound increases the production of the glycopeptide antibiotic, as reported by Beltrametti et al. [77] in the actinobacteria strain *Nonomuraea* sp. Only pyrrolo [1, 2-a] pyrazine-1, 4-dione, hexahydro-3-(2-methylpropyl) out of the six compounds detected from the extract of *Streptomyces* sp. DST119 has been previously reported for its antimicrobial activity [78, 79]. Two of the seven compounds, carbonic acid, 2,2,2-trichloroethyl undec-10-enyl ester and 1-butanol, 2-methyl-acetate from the extract of *Streptomyces flavogriseus* DST52 were reported earlier for their antimicrobial activities [80–82]. Only one compound was determined in the extracts of *Streptomyces cellulosae* DST28 and *Streptomyces* sp. DST116 with a single peak. Di-*n*-octyl phthalate obtained from *Streptomyces cellulosae* DST28 was reported earlier by various researchers for its antimicrobial activity [83, 84], while no activity was reported for 2-methoxy-4,5-diphenyl-6-(2′-phenylethyl)-pyrimidine obtained from the extract of *Streptomyces* sp. DST116 for its antimicrobial activity. Thus, from this study, we conclude that further investigation of the purification of these potent compounds will certainly explicate their efficacy in the pharmaceutical industry. Hence, the usage of freshwater bio-resources can be an ideal source for the isolation of actinobacterial cultures with rare and unique properties that could certainly add to the ever-growing pharmaceutical needs and other biotechnological applications.

## Additional file

**Additional file 1.** Additional tables and figures.
