# Correction to: Bioprospection of actinobacteria derived from freshwater sediments for their potential to produce antimicrobial compounds

**DOI:** 10.1186/s12934-018-0933-8

**Published:** 2018-06-05

**Authors:** Zothanpuia, Ajit Kumar Passari, Vincent Vineeth Leo, Preeti Chandra, Brijesh Kumar, Chandra Nayak, Abeer Hashem, Elsayed Fathi Abd_Allah, Abdulaziz A. Alqarawi, Bhim Pratap Singh

**Affiliations:** 1 Molecular Microbiology and Systematics Laboratory, Department of Biotechnology, Mizoram University, Aizawl, Mizoram 796004 India; 2 SAIF, CSIR-Central Drug Research Institute (CSIR-CDRI), Lucknow, 226012 India; 3 University of Mysore, Manasagangotri, Mysore India; 4 Botany and Microbiology Department, College of Science, King Saud University, P.O. Box 2460, Riyadh, 11451 Saudi Arabia; 5 Mycology and Plant Disease Survey Department, Plant Pathology Research Institute, ARC, Giza, 12511 Egypt; 6 Department of Plant Production, Faculty of Food & Agricultural Sciences, P.O. Box 2460, Riyadh, 11451 Saudi Arabia

## Correction to: Microb Cell Fact (2018) 17:68 10.1186/s12934-018-0912-0

Upon publication of this article [1], it was brought to our attention that Figs. 3, 4 and 5 are incorrectly presented in the original version of the article. The figures were inadvertently swapped in the original submission and published. Figure 3 should be treated as Fig. 5; Fig. 4 should be 3 and Fig. 5 should be Fig. 4.

The corrected figures are given in this erratum (Figs. 3, 4, 5).

**Fig. 3:**
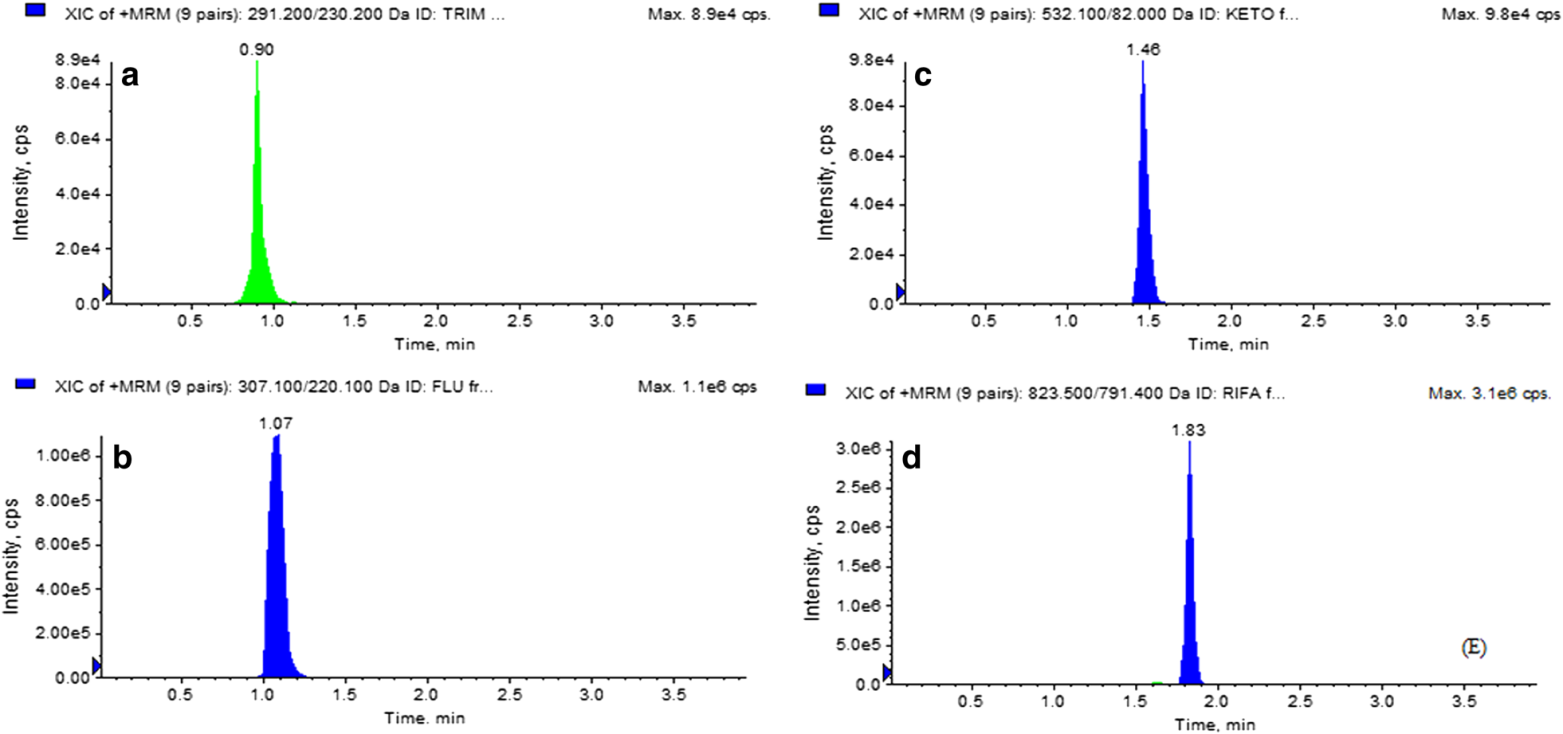
MRM extracted ion chromatogram of reference analyte: **a** trimethoprim, **b** fluconazole, **c** ketoconazole, **d** rifampicin

**Fig. 4:**
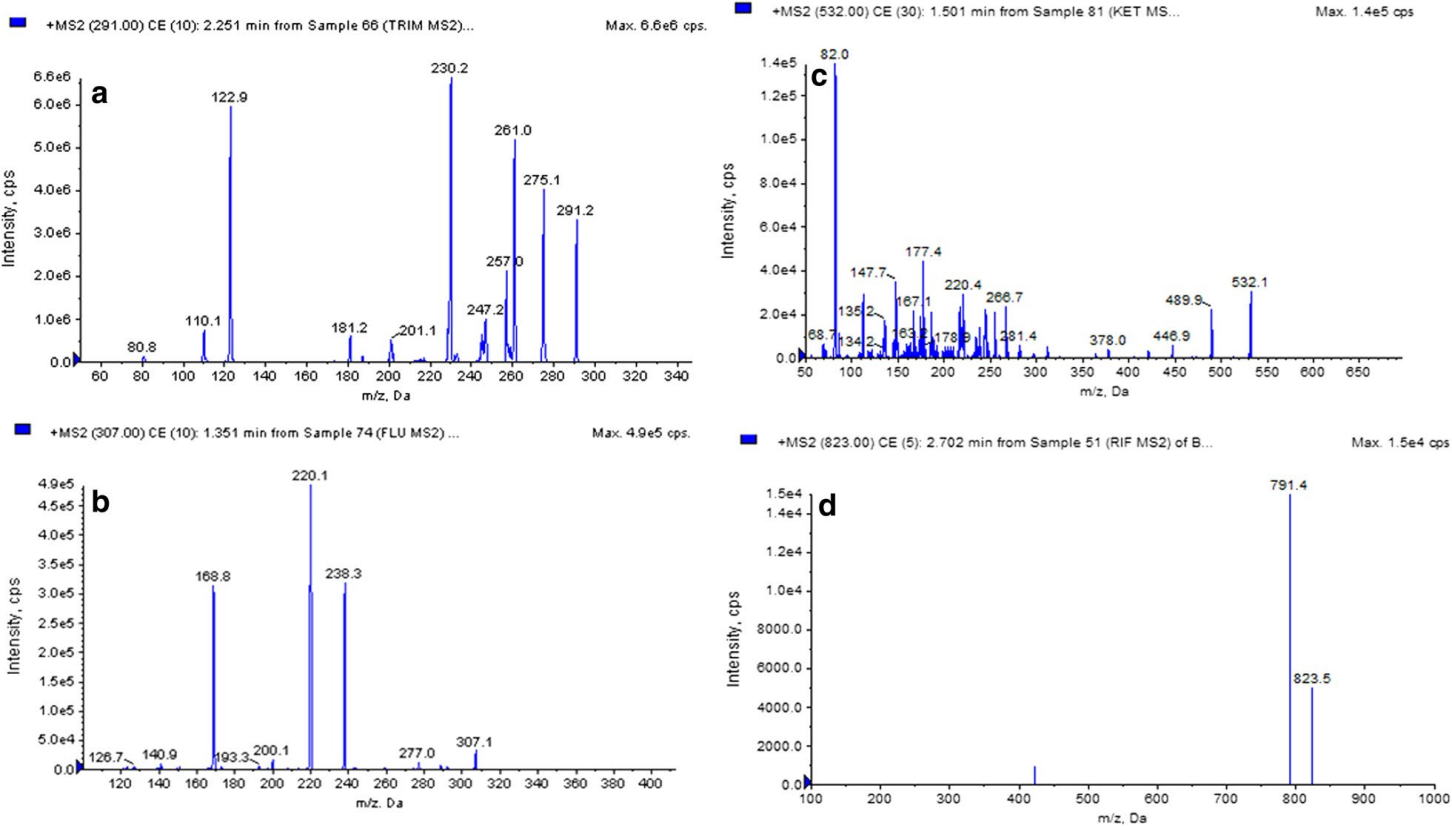
MS/MS spectra of reference analytes; **a** trimethoprim, **b** fluconazole, **c** ketoconazole, **d** rifampicin (as per [19])

**Fig. 5:**
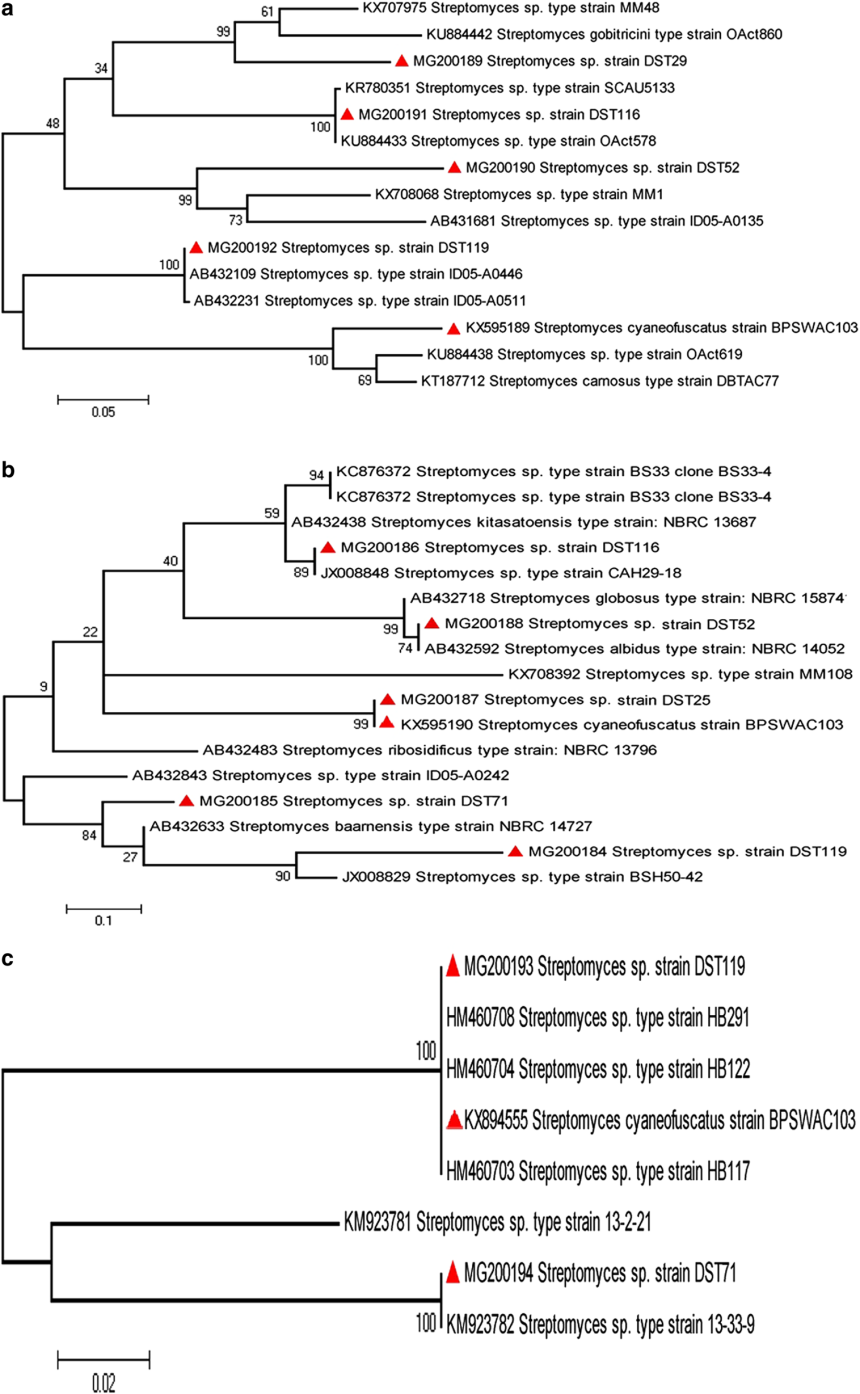
Maximum likelihood (ML) phylogenetic tree constructed using amino acid sequences for **a** PKS type II gene; **b** NRPS gene and **c** phzE gene. The scale bar represents the amino acid changes

Page no. 4 of the original publication under section Detection of antibiotics using UPLC–ESI–MS/MS, last sentence should be “Instrumentation and analytical conditions were performed using the standardized methods as described in our previous paper (Fig. 4) [19].

Similarly, Page no. 8 of original publication under section Detection and quantification of antibiotics using the UPLC-MRM method should be “MS/MS Spectra of standard reference analytes i.e. trimethoprim, fuconazole, ketoconazole and rifampicin showed as Fig. 5 was used from our earlier publication [19].

Page no. 7 of the original publication Figure 3 legend needs to be changed as Fig. 3 MRM extracted ion chromatogram of reference analyte:** a** trimethoprim,** b** fluconazole,** c** ketoconazole,** d** rifampicin.

Page no. 9 of the original publication, Figure 4 should be Fig. 4 MS/MS spectra of reference analytes;** a** trimethoprim,** b** fluconazole,** c** ketoconazole,** d** rifampicin (as per [19]).

Page no. 10 of the original publication, Figure 5 should be Fig. 5 Maximum likelihood (ML) phylogenetic tree constructed using amino acid sequences for** a** PKS type II gene;** b** NRPS gene and** c** phzE gene. The scale bar represents the amino acid changes.
