# Supplementary material for: Bioprospection of actinobacteria derived from freshwater sediments for their potential to produce antimicrobial compounds
Source: Microb Cell Fact. 2018 May 5;17:68. doi: 10.1186/s12934-018-0912-0 (PMC5935920; doi:10.1186/s12934-018-0912-0)
Supplement: Supplementary file 1 — Additional file 1. Additional tables and figures. [file 12934_2018_912_MOESM1_ESM.doc]

**Additional file 1: Table S1.** 68 strains of actinobacteria isolated from three fresh water
 similarity and Genbank accession number

| S. No | Isolate name and strain no. | Closest relative species in BLAST and accession no. | % Similarity | Source | Gene bank Accession number |
| --- | --- | --- | --- | --- | --- |
| 1 | *Promicromonospora sp.*  (DST8) | *Promicromonosporasp*  (KC 550232) | 99% | Tamdil | KM243384 |
| 2 | *Streptomyces violascens* (DST10) | *Streptomyces violascens*  (HQ23834) | 99% | Tlawng | KM405296 |
| 3 | *Streptomyces sp.*  (DST12) | *Streptomyces sp.*  (KJ494303) | 99% | Tamdil | KM405297 |
| 4 | *Streptomyces sp.*  (DST13) | *Streptomyces sp.*  (FJ490543) | 100% | Tamdil | KM405298 |
| 5 | *Streptomyces cyaneofuscatus*  (DST15) | *Streptomyces cyaneofuscatus*  (HG965215) | 99% | Tamdil | KM405300 |
| 6 | *Streptomyces Sp.*  (DST16) | *Streptomyces sp.*  *(KJ889223)* | 99% | Tlawng | KM405301 |
| 7 | *Streptomyces sp.*  (DST17) | *Streptomyces sp.*  (KF770900) | 99% | Tlawng | KM405302 |
| 8 | *Streptomyces sp.*  (DST18) | *Streptomyces sp.*  (EF577245) | 99% | Tamdil | KM405303 |
| 9 | *Streptomyces sp.*  (DST19) | *Streptomyces sp.*  (EF577245) | 99% | Tamdil | KM405304 |
| 10 | *Nocardiopsis sp.*  (DST21) | *Nocardiopsis s.p*  (KJ470127) | 99% | Tamdil | KM405306 |
| 11 | *Prauserella sp.*  (DST22) | *Prauserella sp.*  (KJ574161) | 99% | Tamdil | KM405307 |
| 12 | *Streptomyces sp.*  (DST25) | *Streptomyces sp.*  (FJ492849) | 99% | Tamdil | KM405310 |
| 13 | *Streptomyces cellulosae*  (DST28) | *Streptomyces cellulosae*  (KC429591) | 99% | Tamdil | KM406395 |
| 14 | *Streptomyces flavogriseus*  (DST30) | *Streptomyces flavogriseus*  (KC990785) | 100% | Tamdil | KM406397 |
| 15 | *Saccharopolyspora sp.*  (DST31) | *Saccharopolyspora sp.*  (GUT23669) | 98% | Tamdil | KM406398 |
| 16 | *Nocardiopsis*  (DST32) | *Nocardiopsis*  (KF543090) | 99% | Tamdil | KR703473 |
| 17 | *Streptomyces sp.*  (DST35) | *Streptomyces sp.*  (KM507719) | 98% | Tamdil | KR703474 |
| 18 | *Rhodococus sp.*  (DST38) | *Rhodococus sp.*  (KP128889) | 99% | Tamdil | KR703475 |
| 19 | *Kocuria palustris*  (DST43) | *Kocuria palustris*  (KP324952) | 99% | Tamdil | KR857285 |
| 20 | *Streptomyces pactum*  (DST44) | *Streptomyces pactum*  (KP324952) | 100% | Tamdil | KR857286 |
| 21 | *Nocardiopsis sp.*  (DST46) | *Nocardiopsis sp.*  (KP768309) | 99% | Tamdil | KR857288 |
| 21 | *Streptomyces koyangenesis*  (DST48) | *Streptomyces koyangenesis*  (KM678242) | 100% | Tamdil | KR857290 |
| 23 | *Micrococcus luteus*  (DST49) | *Micrococcus luteus*  (JF303043) | 99% | Tamdil | KR857291 |
| 24 | *Streptomyces sp.*  (DST50) | *Streptomyces sp.*  (KM979603) | 99% | Tamdil | KR857292 |
| 25 | *Rhodococcus sp.*  (DST51) | *Rhodococcus sp.*  (KP128889) | 100% | Tamdil | KR857293 |
| 26 | *Streptomyces flavogriseus*  (DST52) | *Streptomyces flavogriseus*  (GU166435) | 99% | Tamdil | KR857294 |
| 27 | *Streptomyces griseoplanus*  (DST53) | *Streptomyces griseoplanus*  (Hq238386) | 99% | Tamdil | KR857295 |
| 28 | *Streptomyces sp.*  (DST54) | *Streptomyces sp.*  (GU550579) | 99% | Tamdil | KR857296 |
| 29 | *Streptomyces sp.*  (DST56) | *Streptomyces sp.*  (JQ838074) | 99% | Tamdil | KR857298 |
| 30 | *Streptomyces cyaneofuscatus*  (DST57) | *Streptomyces cyaneofuscatus*  (LN824215) | 99% | Tamdil | KR857299 |
| 31 | *Streptomyces somaliensis*  (DST58) | *Streptomyces somaliensis*  (KF973287) | 99% | Tamdil | KR857300 |
| 32 | *Streptomyces cyaneofuscatus*  (DST59) | *Streptomyces cyaneofuscatus*  (LN824215) | 99% | Tamdil | KR857301 |
| 33 | *Streptomyces sp.*  (DST60) | *Streptomyces sp.*  (KM242419) | 99% | Tamdil | KR857302 |
| 34 | *Amycolatopsis sp.*  (DST61) | *Amycolatopsis sp.*  (AF466096) | 99% | Tlawng | KR857303 |
| 35 | *Streptomyces sp.*  (DST62) | *Streptomyces sp.*  (KM979603) | 99% | Tlawng | KR857304 |
| 36 | *Streptomyces sp.*  (DST63) | *Streptomyces sp.*  (KJ494330) | 99% | Tlawng | KR857305 |
| 37 | *Streptomyces* *cyaneofuscatus*  (DST64) | *Streptomyces* *cyaneofuscatus*  (LN824215) | 99% | Tlawng | KR857306 |
| 38 | *Streptomyces lavendulae*  (DST65) | *Streptomyces lavendulae*  (KC626003) | 99% | Tlawng | KR857307 |
| 39 | *Streptomyces olivaceus*  (DST66) | *Streptomyces olivaceus*  (AB184793) | 99% | Tlawng | KR857308 |
| 40 | *Streptomyces griseoplanus*  (DST67) | *Streptomyces griseoplanus*  (HQ238386) | 99% | Tlawng | KR857309 |
| 41 | *Streptomyces violarus*  (DST68) | *Streptomyces violarus*  (NR041116) | 99% | Tlawng | KR857310 |
| 42 | *Streptomyces sp.*  (DST69) | *Streptomyces sp*.  (GU550579) | 99% | Tlawng | KR857311 |
| 43 | *Streptomyces* rubiginosohelvolus  (DST70) | *Streptomyces* rubiginosohelvolus  (KJ632658) | 99% | Tlawng | KR857312 |
| 44 | *Streptomyces albidoflavus*  (DST71) | *Streptomyces albidoflavus*  (KP122209) | 99% | Tlawng | KR857313 |
| 45 | *Streptomyces rubiginosohelvolus*  (DST72) | *Streptomyces rubiginosohelvolus*  (KJ632658) | 100% | Tlawng | KR857314 |
| 46 | *Streptomyces atratus*  (DST73) | *Streptomyces atratus*  (KC462521) | 99% | Tlawng | KR857315 |
| 47 | *Streptomyces atroolivaceus*  (DST74) | *Streptomyces atroolivaceus*  (HQ831417) | 100% | Tlawng | KR857316 |
| 48 | *Streptomyces koyangenesis*  (DST75) | *Streptomyces koyangenesis*  (KM678242) | 99% | Tlawng | KR857317 |
| 49 | *Streptomyces qancidicus*  (DST76) | *Streptomyces qancidicus*  (KP792994) | 99% | Tlawng | KR857318 |
| 50 | *Streptomyces sp.*  (DST86) | *Streptomyces sp.*  (KT232313) | 99% | Tuirial | KT232313 |
| 51 | *Micrococcus luteus*  (DST87) | *Micrococcus luteus*  (KM37857) | 99% | Tuirial | KT232314 |
| 52 | *Micrococcus* sp.  (DST88) | *Micrococcus sp.*  (LN846826) | 99% | Tuirial | KT232315 |
| 53 | *Saccharopolyspora sp.* (DST89) | *Saccharopolyspora sp.*  (JQ885595) | 99% | Tuirial | KT232316 |
| 54 | *Nocardiopsis*  (DST95) | *Nocardiopsis*  ([KF270095](http://www.ncbi.nlm.nih.gov/nucleotide/525330037?report=genbank&log$=nucltop&blast_rank=2&RID=G3ZWVJRT014)) | 99% | Tuirial | KT429605 |
| 55 | *Streptomyces albidoflavus*  (DST96) | *Streptomyces albidoflavus*  ([KT385695](http://www.ncbi.nlm.nih.gov/nucleotide/982894644?report=genbank&log$=nucltop&blast_rank=5&RID=G3Z0GX7B014)) | 99% | Tuirial | KT429606 |
| 56 | *Saccharopolyspora* sp. (DST97) | *Saccharopolyspora sp.*  ([GU723669](http://www.ncbi.nlm.nih.gov/nucleotide/293629561?report=genbank&log$=nucltop&blast_rank=3&RID=G3Z1BAV8015)) | 98% | Tuirial | KT429607 |
| 57 | *Saccharopolyspora sp.* (DST98) | *Saccharopolyspora sp*  ([GU723669](http://www.ncbi.nlm.nih.gov/nucleotide/293629561?report=genbank&log$=nucltop&blast_rank=3&RID=G40RNPJP01R))*.* | 98% | Tuirial | KT429608 |
| 58 | *Streptomyces cyaneofuscatus*  (DST99) | *Streptomyces cyaneofuscatus*  ([KR857299](http://www.ncbi.nlm.nih.gov/nucleotide/848832335?report=genbank&log$=nucltop&blast_rank=2&RID=G4045R45014)) | 99% | Tuirial | KT429609 |
| 59 | *Streptomyces albidoflavus*  (DST100) | *Streptomyces albidoflavus*  (KT385695) | 99% | Tuirial | KT429610 |
| 60 | *Streptomyces albidoflavus*  (DST102) | *Streptomyces albidoflavus*  (KT385695) | 99% | Tuirial | KT429612 |
| 61 | *Streptomyces sp.*  (DST104) | *Streptomyces sp.*  (HM036677) | 99% | Tuirial | KT429614 |
| 62 | *Nocardiopsis*  (DST105) | *Nocardiopsis*  ([KT025849](http://www.ncbi.nlm.nih.gov/nucleotide/946776454?report=genbank&log$=nucltop&blast_rank=1&RID=G401M46J01R)) | 100% | Tuirial | KT429615 |
| 63 | *Streptomyces atroolivaceus*  (DST106) | *Streptomyces atroolivaceus*  (KX130875) | 99% | Tuirial | KT429616 |
| 64 | *Streptomyces sp.*  (DST116) | *Streptomyces sp.*  (KM368927) | 99% | Tuirial | KY077681 |
| 65 | *Nocardiopsis sp.*  (DST117) | *Nocardiopsis sp.*  (KX502849) | 99% | Tuirial | MF536299 |
| 66 | *Streptomyces griseus*  (DST118) | *Streptomyces griseus*  (YIM130689) | 100% | Tuirial | MF536300 |
| 67 | *Streptomyces sp*  (DST119) | *Streptomyces sp*  (EU734615) | 99% | Tuirial | MF536301 |
| 68 | *Streptomyces fulvissimus*  (DST120) | *Streptomyces fulvissimus*  (KX714721) | 100% | Tuirial | MF536302 |

**Additional file 1: Table S2.** Antimicrobial activity and biosynthetic gene detection of 68 actinobacteria isolates

| Isolate code | Antibacterial properties | | | | | Yeast | Biosynthetic genes | | |
| --- | --- | --- | --- | --- | --- | --- | --- | --- | --- |
|  | *E. coli* | *P. aeruginosa* | *S. aureus* | *M. luteus* | *B subtilis* | *C. albicans* | PKS-II | NRPS | *phe*Z |
| DST8 | 14.5±0.05 | 09.0±0.05 | 05.6±0.20 | - | - | 12.2±0.10 |  | - | - |
| DST10 | 10.4±0.05 | 11.00±0.01 | 08.34±0.03 | - | 13.2±0.20 | 11.0±0.10 | - | - | - |
| DST12 | 08.0±0.30 | 04.0±0.10 | 08.0±0.26 | - | - | - | - | - | - |
| DST13 | 09.50±0.01 | 10.5±0.50 | - | - | - | 9.00±0.50 | + | + | - |
| DST15 | 09.25±0.05 | 12.10±0.01 | 9.43±0.03 | 05.2±0.10 | - | 10.5±0.50 | + | + | - |
| DST16 | 10.06±0.06 | 08.00±0.10 | 10.1±0.01 | 13.4±0.10 | 10.4±0.03 |  | + | + | - |
| DST17 | 10.35±0.05 | - | 09.00±0.05 | 05.4±0.20 | 13.5±0.10 | 12.1±0.10 | - | - | - |
| DST18 | 08.0±0.20 | 09.0±0.23 | - | 11.2±0.10 | 14.5±0.30 | 13.8±0.30 | - | - | - |
| DST19 | 13.5±0.05 | 09.0±0.10 | 12.3±0.10 | - | 12.3±0.10 | 13.8±0.20 | - | - | - |
| DST21 | 10.62±0.02 | 10.0±0.10 | 08.62±0.02 | - | - | - | - | - | - |
| DST22 | 10.5±0.50 | 09.0±0.50 | - | - | 9.00±0.50 | - | - | - | - |
| DST25 | 9.41±0.03 | 12.00±0.06 | 09.00±0.06 | 13.2±0.10 | 12.5±0.20 | 12.5±0.20 | + | + | - |
| DST28 | 09.50±0.01 | 10.00±0.10 | 10.00±0.10 | 12.6±0.10 | 12.5±0.10 | 11.5±0.50 | - | - | - |
| DST30 | 10.20±0.03 | 06.40±0.07 | 10.50±0.01 |  | 14.8±0.30 | 12.8±0.10 | - | + | - |
| DST31 | 12.00±0.09 | 12.42±0.02 | 09.20±0.05 | - | 12.5±0.20 | 12.2±0.10 | - | - | - |
| DST32 | 14.00±0.10 | 10.00±0.05 | 07.5±0.20 | - | 13.0±0.20 | 12.0±0.05 | - | - | - |
| DST35 | 10.0±0.20 | 09.0±0.50 | - | 12.5±0.10 | 10.4±0.05 | 12.2±0.10 | - | - | - |
| DST38 | 10.0±0.20 | 07.8±0.20 | 10±0.10 | - | 13.0±0.30 | 12.5±0.15 | - | - | - |
| DST43 | 10.35±0.05 | 08.20±0.05 | 09.65±0.02 | - | - | - | + | + | - |
| DST44 | 8.50±0.15 | 08.0±0.20 | 07.20±0.10 | 06.4±0.30 | 13.4±0.10 | - | + | + | - |
| DST46 | 12.0±0.20 | - | - | 09.8±0.10 | 12.2±0.30 | 11.0±0.10 | - | + | - |
| DST48 | 9.2±0.10 | 09.4±0.10 | - | 11.2±0.20 | 13.4±0.10 | 13.2±0.20 | - | + | - |
| DST49 | 12.5±0.05 | 10.0±0.03 | 07.2±0.10 | 12.8±0.10 |  | 14.5±0.20 | - | + | - |
| DST50 | 14±0.05 | - | 12.1±0.30 | 07.2±0.30 | 12.4±0.10 | 14.2±0.20 | - | + | + |
| DST51 | 10.4±0.10 | 09.5±0.04 | 07.5±0.05 | 14.3±0.20 | 13.5±0.05 | 14.2±0.05 | - | + | - |
| DST52 | 15±0.010 | 08.0±0.10 | 08±0.050 | 10.8±0.10 | 08.4±0.01 | 15.5±0.05 | + | + | - |
| DST53 | 09.5±0.10 | 10.8±0.30 | 05.8±0.30 | - | 07.6±0.04 | 12.2±0.20 | + | + | - |
| DST54 | 14.5±0.10 |  | 04.5±0.05 | 05.1±01 | 06.8±0.10 | 10.2±0.20 | + | + | + |
| DST56 | 11.85±0.35 | 9.00±0.50 | - | - | 12.4±0.40 | 10.4±0.10 | + | + | + |
| DST57 | 15.95±0.05 | 09.0±0.35 | 06±0.10 | - | 15.6±0.30 | 15.2±0.10 | + | + | + |
| DST58 | 10.2±0.10 | - | - | 12.2±0.30 | 10.8±0.10 | 12.4±0.20 | + | + | + |
| DST59 | 13.0±0.1 | 06.0±0.30 | - | - | 10.4±0.10 | 11.8±0.10 | + | + | - |
| DST60 | 13.5±0.15 | 08.0±0.25 | - | - | 12.0±0.10 | 14.3±0.05 | - | + | + |
| DST61 | 15.0±0.05 | 10.0±0.35 | - | - | - | 13.4±0.20 | + | + | - |
| DST62 | 08.2±0.00 | 12.1±0.20 | - | 10.8±0.10 | 06.8±0.20 | 13.0±0.30 | - | + | - |
| DST63 | 11.5±0.10 | 9.00±0.35 | - | 12.4±0.10 | 11.2±0.50 | 11.5±0.20 | - | + | - |
| DST64 | 09.8±0.20 | 09.8±0.20 | 12.2±0.20 | - | 9.8±0.10 | 12.1±0.40 | - | + | + |
| DST65 | 08.5±0.05 | 09.0±0.50 | - | - | 9.00±0.50 | - | + | + | - |
| DST66 | 10.5±0.15 | 05.0±0.10 | 07.4±0.10 | - | 12.5±0.01 | 10.4±0.10 | - | + | + |
| DST67 | 07.5±0.20 | 10.5±0.20 | - | - | 10.8±0.20 | 10.4±0.20 | - | + | + |
| DST68 | 07.4±0.05 | 10.1±0.10 | - | - | 09.4±0.40 | 12.0±0.20 | - | - | - |
| DST69 | 14.0±0.15 | 08.0±0.10 | - | - | 11.9±0.10 | 12.2±0.15 | + | + | - |
| DST70 | 09.4±0.10 | 10.4±0.30 | - | - | 07.4±0.30 | 10.1±0.40 | - | + | - |
| DST71 | 13.0±0.30 | 08.0±0.50 | 06.6±0.40 | 06.2±0.20 | 15.9±0.20 | 13.8±0.10 | - | + | + |
| DST72 | 10.2±0.30 | 10.8±0.4 | 09.8±0.10 | 07.8±0.10 | - | 12.2±0.30 | - | + | - |
| DST73 | 7.95±0.05 | 10.5±0.50 | - | - | - | - | - | + | - |
| DST74 | 12.5±0.15 | 09.0±0.50 | 05.2±0.30 | - | 09.4±0.04 | 13.2±0.05 | + | + | + |
| DST75 | 09.10±0.16 | 09.23±0.03 | 12.50±0.10 | - | 15.0±0.20 | - | - | + | - |
| DST76 | 10.00±0.10 | 10.63±0.03 | 08.37±0.02 | - | 15.2±0.30 | 13.4±0.20 | + | + | + |
| DST86 | 10.50± 0.05 | 08.30±0.05 | 09.10±0.01 | - | - | - | - | - | - |
| DST87 | 7.95±0.05 | 7.05±0.05 | 04.0±0.10 | - | 15.0±0.30 | - | - | - | - |
| DST88 | 14.0±0.25 | 08.5±0.05 | - | - | - | - | - | - | - |
| DST89 | 10.0±0.10 | 07.8±0.15 | 10.06±0.05 | - | - | - | - | - | - |
| DST95 | 8.95±0.05 | 09±0.32 | 7.95±0.05 | - | 10.5±0.30 | 12.4±0.10 | + | + | - |
| DST96 | 14.5±0.10 | 09±0.10 | - | 09.2±0.10 | 14.5±0.20 | 11.8±0.10 | - | + | - |
| DST97 | 10.13±0.05 | 09.18±0.02 | 10.0±0.10 | - | - | - | - | + | - |
| DST98 | 9.00±0.50 | 10.1±0.30 | 10.1±0.20 | - | - | 11.5±0.05 | - | - | - |
| DST99 | 15.5±0.20 | 7.5±0.30 | 10.4±0.30 | - | 12.2±0.10 | 10.6±0.20 | + | + | + |
| DST100 | 13.0±0.40 | 9.8±0.05 | 10.6±0.30 | - | 07.3±0.01 | 12.5±0.10 | - | + | - |
| DST102 | 12.0±0.10 | 05.0±0.30 | 15.0±0.20 | - | 10.2±0.10 | 12.4±0.30 | - | + | + |
| DST104 | 13.5±0.10 | - | 5.60±0.01 | 05.3±0.50 | 15.0± 0.50 | 11.5±0.20 | - | + | - |
| DST105 | 13.00±0.05 | 05.0±0.40 | - | - | 08.4±0.20 | 11.5±0.20 | + | + | - |
| DST106 | 14.0±0.05 | 08.5±0.10 | 05.4±0.10 |  | 12.6±0.10 | 12.3±0.05 | + | - | + |
| DST116 | 0.09±0.30 | 05.8±0.20 | 09.2±0.25 | 18.8±0.10 | 14.4±0.20 | 12.9±0.05 | + | + | + |
| DST117 | 12.5±0.30 | 12.2±0.10 | - | - | 11.6±0.10 | 11.5±0.20 | - | + | - |
| DST118 | 08.00±0.20 | 07.90±0.10 | 03.0±0.30 | 10.8±0.10 | 14.2±0.10 | 12.2±0.20 | + | + | + |
| DST119 | 08.10±0.10 | 07.0±0.10 | 08.00±0.10 | 14.3±0.10 | 14.2±0.10 | 13.1±0.10 | + | + | + |
| DST120 | 14.5±0.15 | 10.0±0.05 | - | - | 12.4±0.20 | 14.8±0.20 | + | + | + |

**Additional file 1: Table S3. Volatile organic compounds determined using GC-MS**

| Sl no. | Compound name | Retention time (min) | Area (%) | Molecular Formula | Molecular weight (MW) | Quality (%) | Activity | Reference |
| --- | --- | --- | --- | --- | --- | --- | --- | --- |
| ***Streptomyces sp*. strain DST25** | | | | | | | | |
| 1 | Valine | 15.383 | 23.413 | C_5_H_11_O_2_N | 117 | 75 | antimicrobial and cytotoxic activity | Lee et al., 2014 |
| 2 | Glutaraldehyde | 15.668 | 50.075 | C_5_H_8_O_2_ | 100 | 80 | disinfectant, antimicrobial | Lerones et al., 2004; Hill et al., 1991 |
| 3 | D-Leucine | 16.958 | 11.942 | C_6_H_13_O_2_N | 131 | 80 | antibacteria | Fox et al., 1944; Ahmad et al., 2014 |
| 4 | 3,3-Dimethyl-4-methylamino-butan-2-one | 17.458 | 3.585 | C_7_H_15_ON | 129 | 86 | antimicrobial | Dineshkumar et al., 2017 |
| 5 | Heptanal | 17.799 | 5.813 | C_7_H_14_O | 114 | 84 | antimicrobial | Al-Wathnani et al., 2012 |
| 6 | Cyclopropane, 1-butyl-2-(2-methylpropyl)- | 22.390 | 5.172 | C_11_H_22_ | 154 | 80 | no activity found | **-** |
| ***Streptomyces cellulosae* strain DST28** | | | | | | | | |
| 7 | Di-N-Octyl phthalate | 29.673 | 100 | C_24_H_38_0_4_ | 390 | 85 | Antimocrobial | Philip et al., 2011; Shafaghat et al., 2012) |
| ***Streptomyces flavogriseus* strain DST52** | | | | | | | | |
| 8 | Heptacosanoic acid, 25-methyl-, methyl ester | 20.235 | 13.177 | C_29_H_58_O_2_ | 438 | 81 | larvicidal | Balasubramani et al., 2015 |
| 9 | 2-Hexene, 1-butoxy-, (e)- | 20.495 | 13.827 | C_10_H_20_O | 156 | 69 | no activity reported | - |
| 10 | Carbonic acid, 2,2,2-trichloroethyl undec-10-enyl ester | 21.445 | 49.782 | C_14_H_23_O_3_C_l3_ | 344 | 70 | antibacteria | Musini et al., 2013 |
| 11 | 1,6;2,3-Dianhydro-4-deoxy-.beta.-d-ribo-hexopyranose | 21.880 | 12.522 | C_6_H_8_O_3_ | 128 | 71 | anticancer | Zhou et al., 2017 |
| 12 | 1-Butanol, 2-methyl-, acetate | 22.290 | 2.313 | C_7_H_14_O_2_ | 130 | 61 | antimicrobial | Ezra and Strobel. 2003 Ezra et al., 2004 |
| 13 | D-Erythro-pentose, 2-deoxy- | 22.350 | 6.155 | C_5_H_10_O_4_ | 134 | 60 | no activity reported | - |
| 14 | 2H-Pyran-2-methanol, 3-(benzoyloxy)-3,6-dihydro-6-methoxy-, benzoate | 27.778 | 2.224 | C_21_H_2_0O_6_ | 368 | 76 | no activity reported | - |
| ***Streptomyces albidoflavus* strain DST71** | | | | | | | | |
| 15 | 2-Thiophenecarboxylic acid, 5-(1,1-dimethylethoxy)- | 15.838 | 8.764 | C_9_H_12_O_3_S | 200 | 85 | antimicrobial | Al-Wathnani et al., 2012; Perveen and Alwathnani. 2013 |
| 16 | Hexanal | 15.943 | 23.187 | C_6_H_12_O | 100 | 82 | antimicrobial | Rattanakom and Yasurin 2014; Sitarek et al., 2017 |
| 17 | Glutaraldehyde | 16.208 | 4.578 | C_5_H_8_O_2_ | 100 | 82 | disinfectant, antimicrobial | Lerones et al., 2004; Hill et al., 1991 |
| 18 | Oxirane, 2-butyl-3-methyl-, cis | 16.808 | 5.268 | C_7_H_14_O | 114 | 79 | no activity reported | - |
| 19 | Pentadecylamine | 17.013 | 5.176 | C_15_H_33_N | 227 | 81 | antimicrobial | Kabara et al., 1972 |
| 20 | Azacyclodecan-5-ol | 17.408 | 5.820 | C_9_H_19_ON | 157 | 84 | no activity reported | - |
| 21 | Heptanal | 17.433 | 7.692 | C_7_H_14_O | 114 | 88 | antimicrobial | Al-Wathnani et al., 2012 |
| 22 | 3,3-Dimethyl-4-methylamino-butan-2-one | 17.548 | 5.962 | C_7_H_15_ON | 129 | 84 | antimicrobial | Dineshkumar et al., 2017 |
| 23 | Glutaraldehyde | 17.748 | 5.582 | C_5_H_8_O_2_ | 100 | 82 | antimicrobial | Lerones et al., 2004; Hill et al., 1991 |
| 24 | L-Argininic acid | 20.384 | 4.491 | C_6_H_13_O_3_N_3_ | 175 | 73 | antimicrobial | Sepahi et al., 2017 |
| 25 | N-(4-Chlorobenzenesulfonyl)azetidin-3-one | 20.645 | 6.772 | C_9_H_8_O_3_N | 245 | 70 | No activity reported | - |
| 26 | 1,3,5-Triazaadamantane | 21.305 | 7.769 | C_7_H_13_N_3_ | 139 | 72 | No activity reported | - |
| 27 | 2,4,6-Cycloheptatrien-1-one, 3,5-bis-trimethylsilyl- | 28.128 | 3.529 | C_13_H_22_OSi_2_ | 250 | 73 | antioxidant, antimicrobial | Ramakrishnan and Venkataraman . 2011; Foo et al., 2017 |
| 28 | 1,1,1,3,5,5,5-Heptamethyltrisiloxane | 28.892 | 5.411 | C_7_H_22_O_2_Si_3_ | 222 | 81 | antimicrobial | Dehpour et al., 2011 |
| ***Streptomyces sp*. strain DST116** | | | | | | | | |
| 29 | 2-Methoxy-4,5-diphenyl-6-(2'-phenylethyl)pyrimidine | 28.858 | 100 | C_25_H_22_ON_2_ | 366 | 65 | No activity reported | - |
| ***Streptomyces sp*. strain DST119** | | | | | | | | |
| 30 | 1-Carbahexaborane(7) | 21.435 | 3.218 | CH_7_B_5_ | 74 | 70 | no activity reported | - |
| 31 | Pyrrolo[1,2-a]Pyrazine-1,4-dione, hexahydro-3-(2-methylpropyl)- | 21.535 | 21.466 | C_11_H_18_O_2_N_2_ | 210 | 59 | Antimicrobial, antioxidant | Sheoran et al., 2015; Melo et al., 2014; Durai et al., 2013, Jinfeng et al., 2017 |
| 32 | L-Alanine, n-(Cyclohexylcarbonyl)-, Decyl ester | 22.105 | 3.030 | C_20_H_37_O_3_N | 399 | 63 | no activity reported | - |
| 33 | Chlormezanone | 22.301 | 25.682 | C_11_H_12_O_3_N | 273 | 49 | anxiolytic, muscle relaxant | Asif. 2015 |
| 34 | L-Alanine, n-(cyclohexylcarbonyl)-, undec-10-enyl ester | 23.132 | 3.935 | C_21_H_37_O_3_N | 351 | 67 | no activity reported | - |
| 35 | 2-Benzylthio-8-methyl-7-phenylpyrano[2,3-f]benzoxazol-6(h)-one | 30.739 | 42.669 | C_24_H_17_O_3_NS | 399 | 77 | no activity reported | - |

**Additional file 1: Table S4**. LC-MS/MS optimized parameters

| Analytes | *rt* (min) | Q1 | Q3 | Ion species | DP | EP | CE | CXP |
| --- | --- | --- | --- | --- | --- | --- | --- | --- |
| Trimethoprim | 0.9 | 291.2 | 231.2 | [M+H]^+^ | 149 | 6 | 33 | 10 |
| Fluconazole | 1.07 | 307.1 | 220.1 | [M+H]^+^ | 59 | 10 | 27 | 8 |
| Ketoconazole | 1.46 | 532.1 | 82 | [M+H]^+^ | 106 | 10 | 68 | 10 |
| Rifamycin | 1.83 | 823.5 | 791.4 | [M+H]^+^ | 53 | 9 | 24 | 19 |


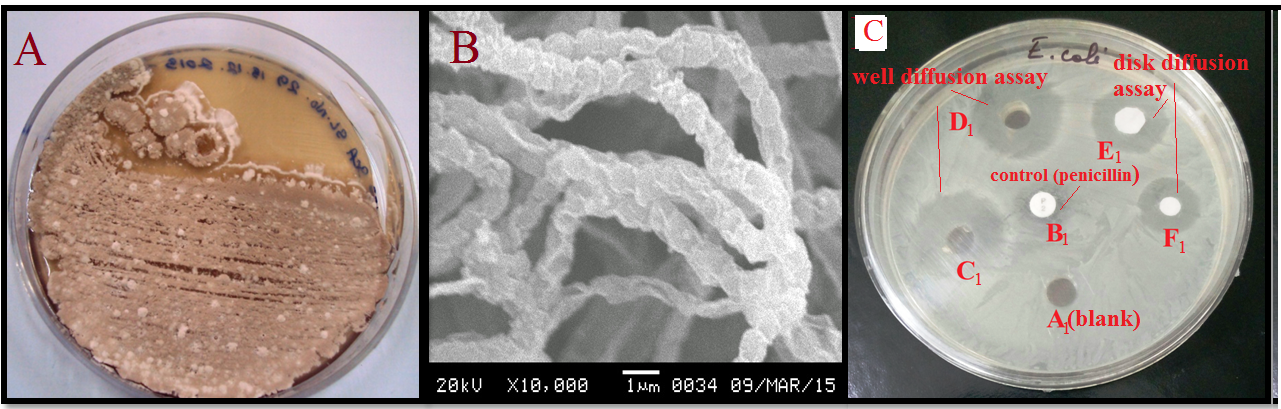


**Additional file 1: Fig.S1** A. Morphological appearance of DST25, B. spore chain morphology of DST25 C. antimicrobial activity of DST25 at different concentrations, D. Comparison of disk diffusion assay and well diffusion assay


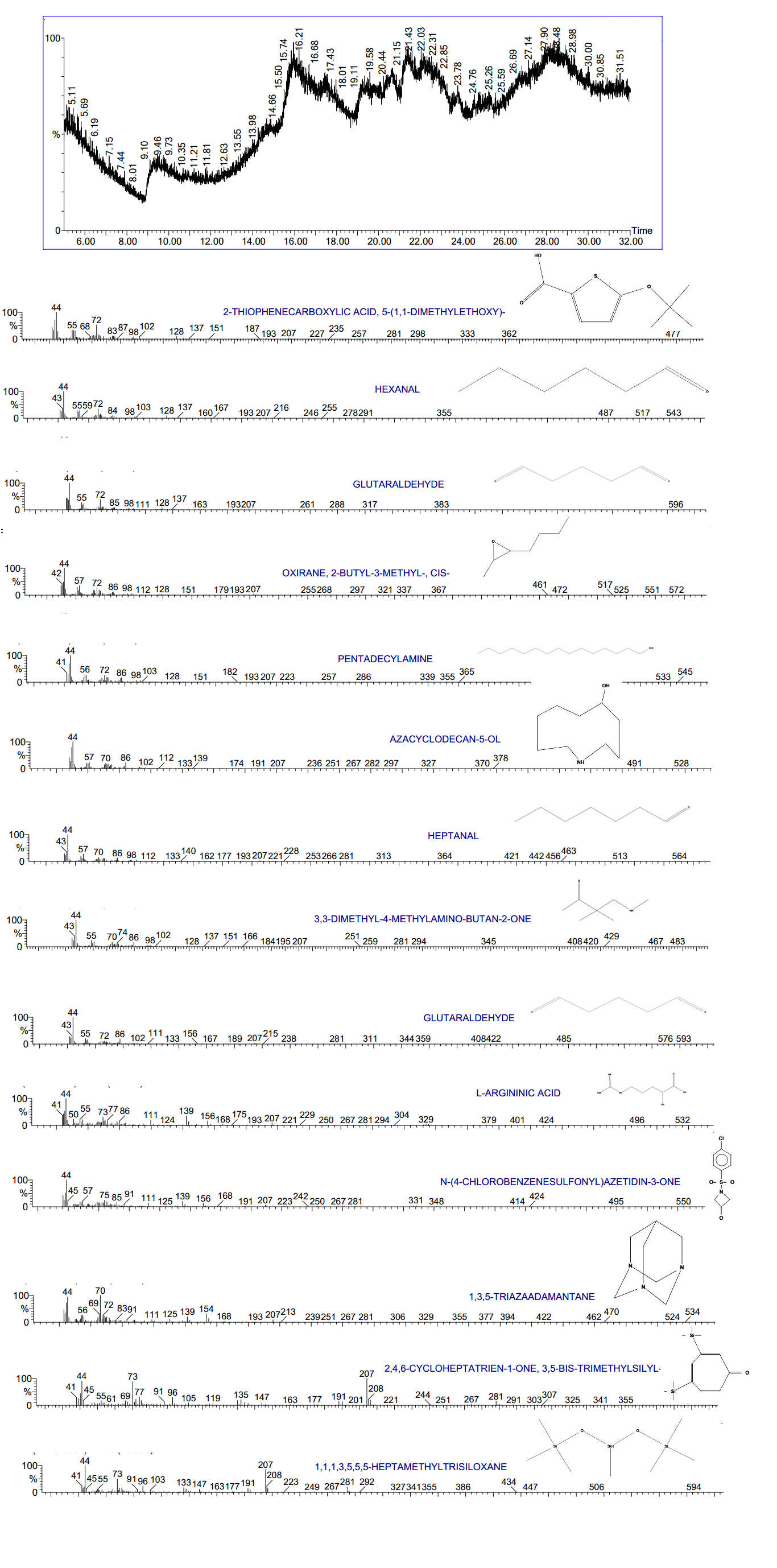


**Fig.S2** VOCs determined using GC/MS in isolate *Streptomyces albidoflavus* strain DST71

**
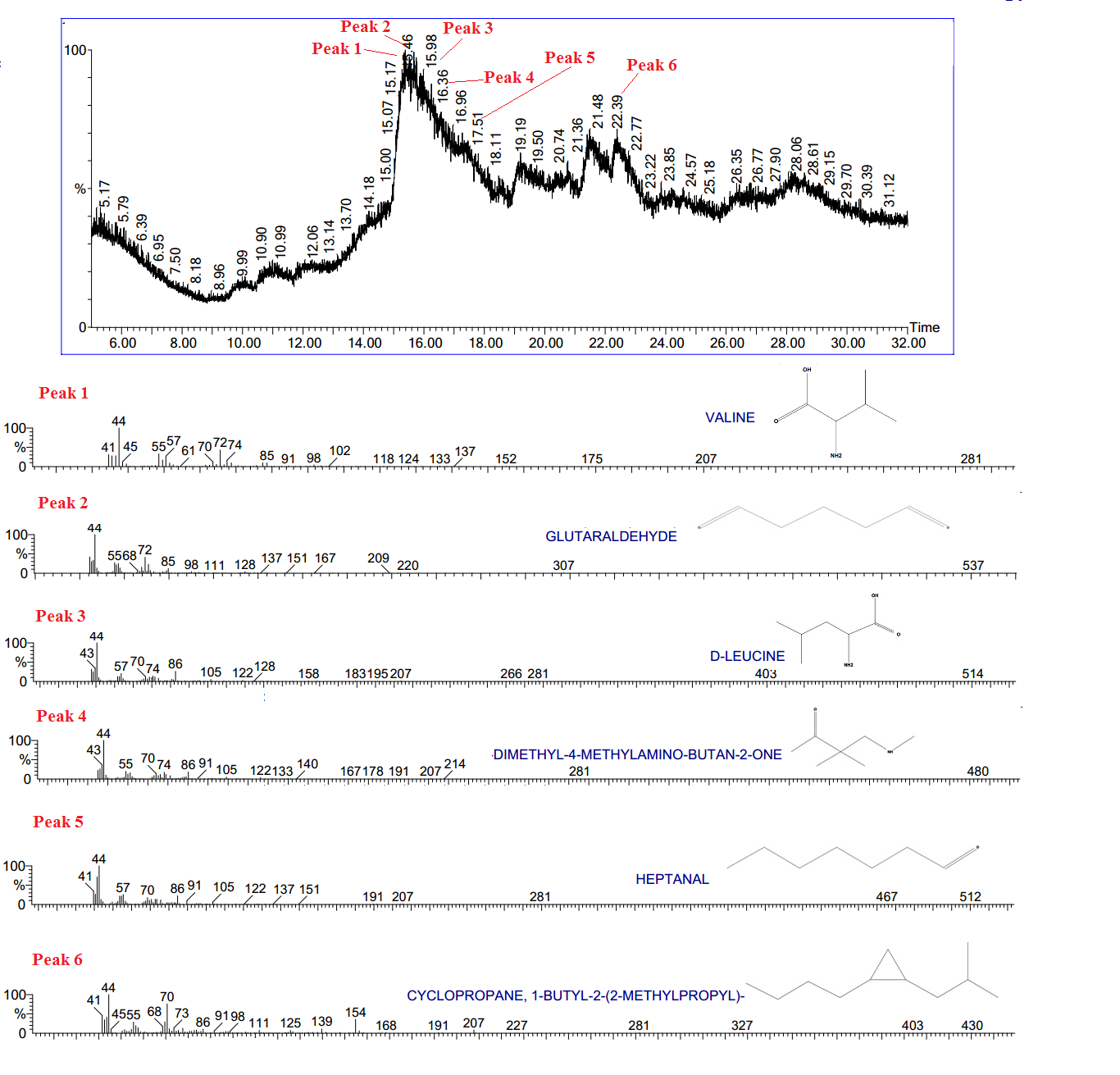
**

**Additional file 1: Fig.S3** VOCs determined using GC/MS in isolate *Streptomyces* sp. strain DST25


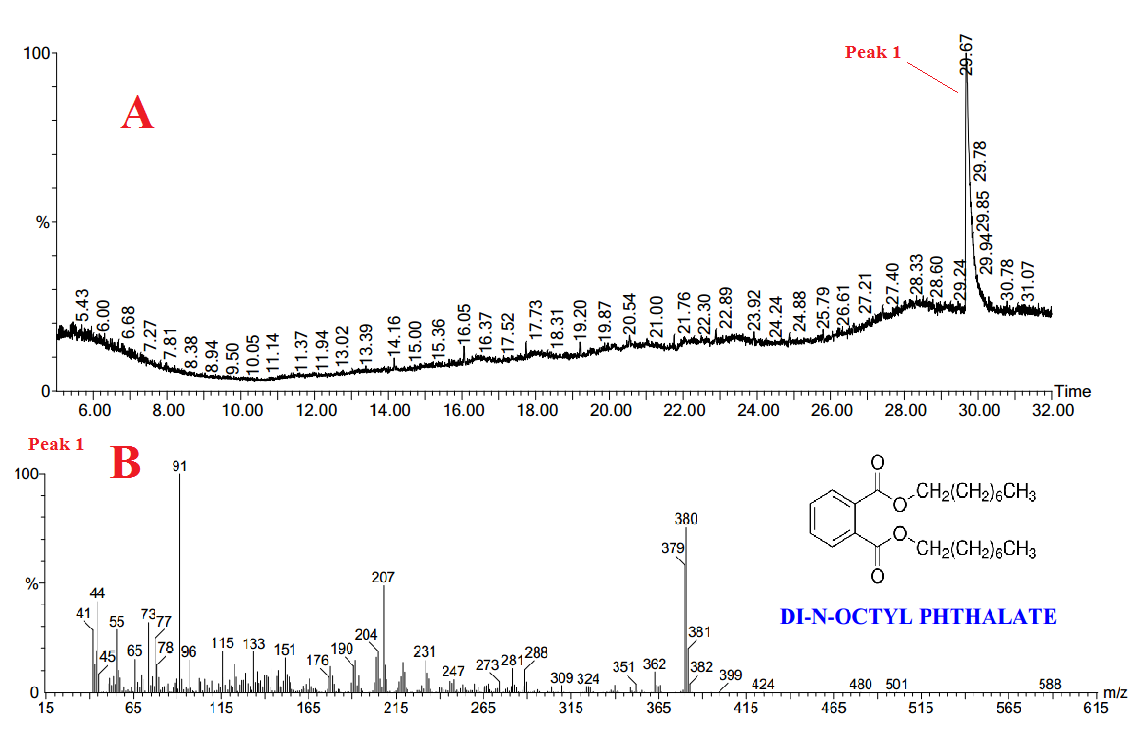


**Additional file 1: Fig.S4** VOCs determined using GC/MS in isolate *Streptomyces cellulosae* strain DST28


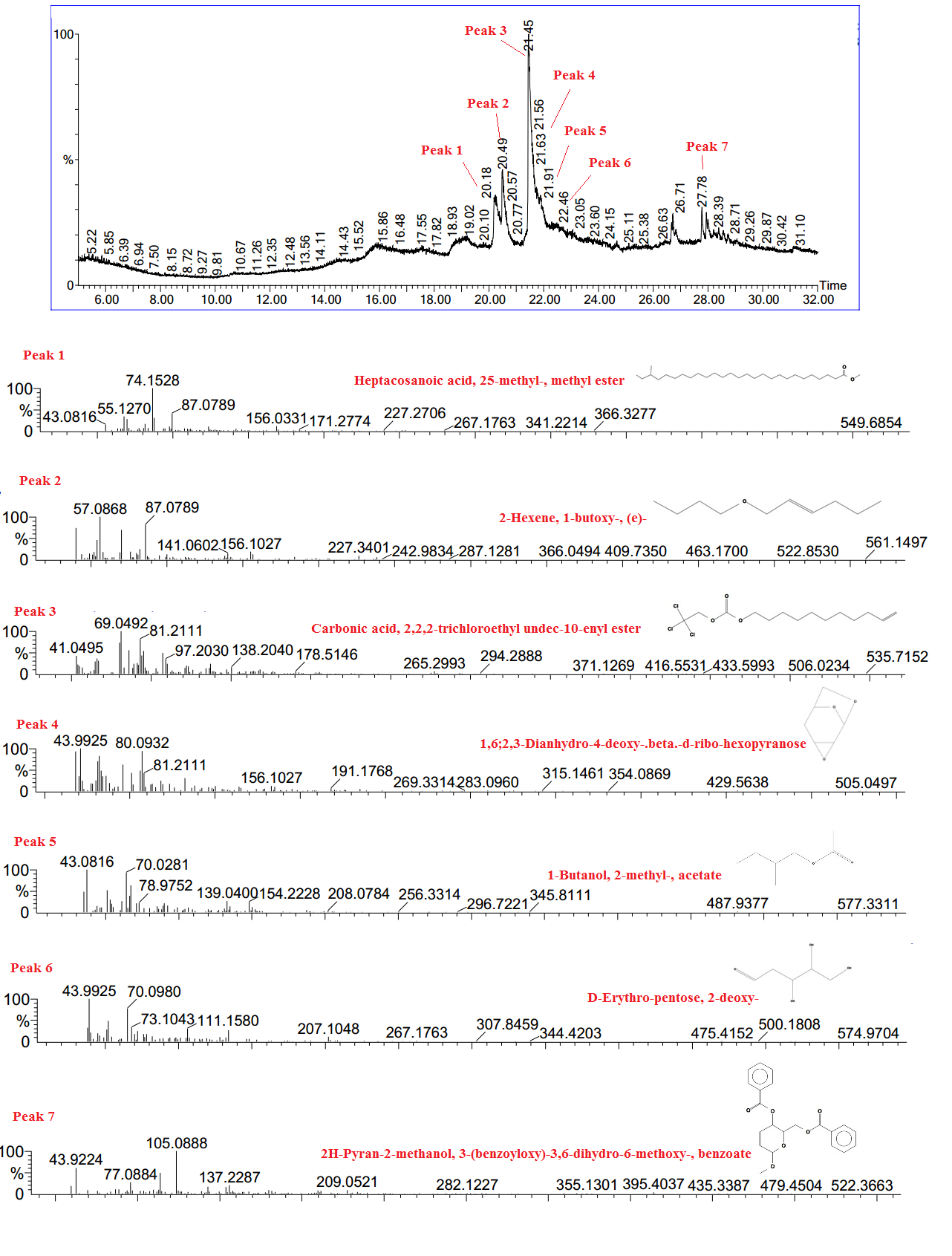


**Additional file 1: Fig.S5** VOCs determined using GC/MS in isolate *Streptomyces flavogriseus* strain DST52


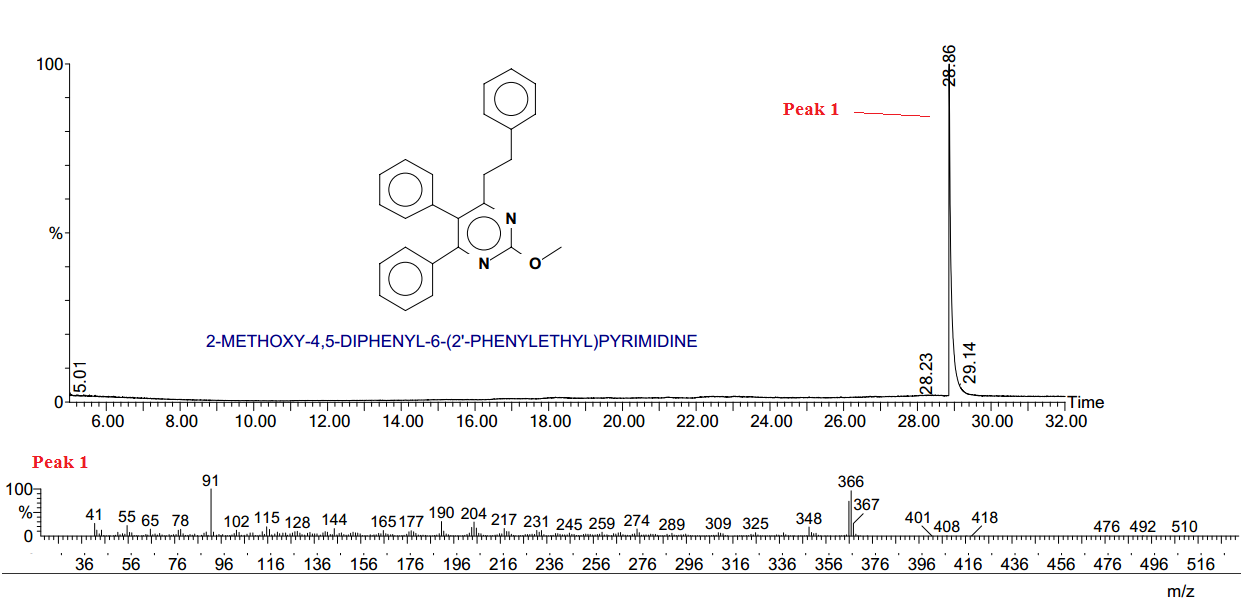


**Additional file 1: Fig.S6** VOCs determined using GC/MS in isolate *Streptomyces* sp. strain DST116


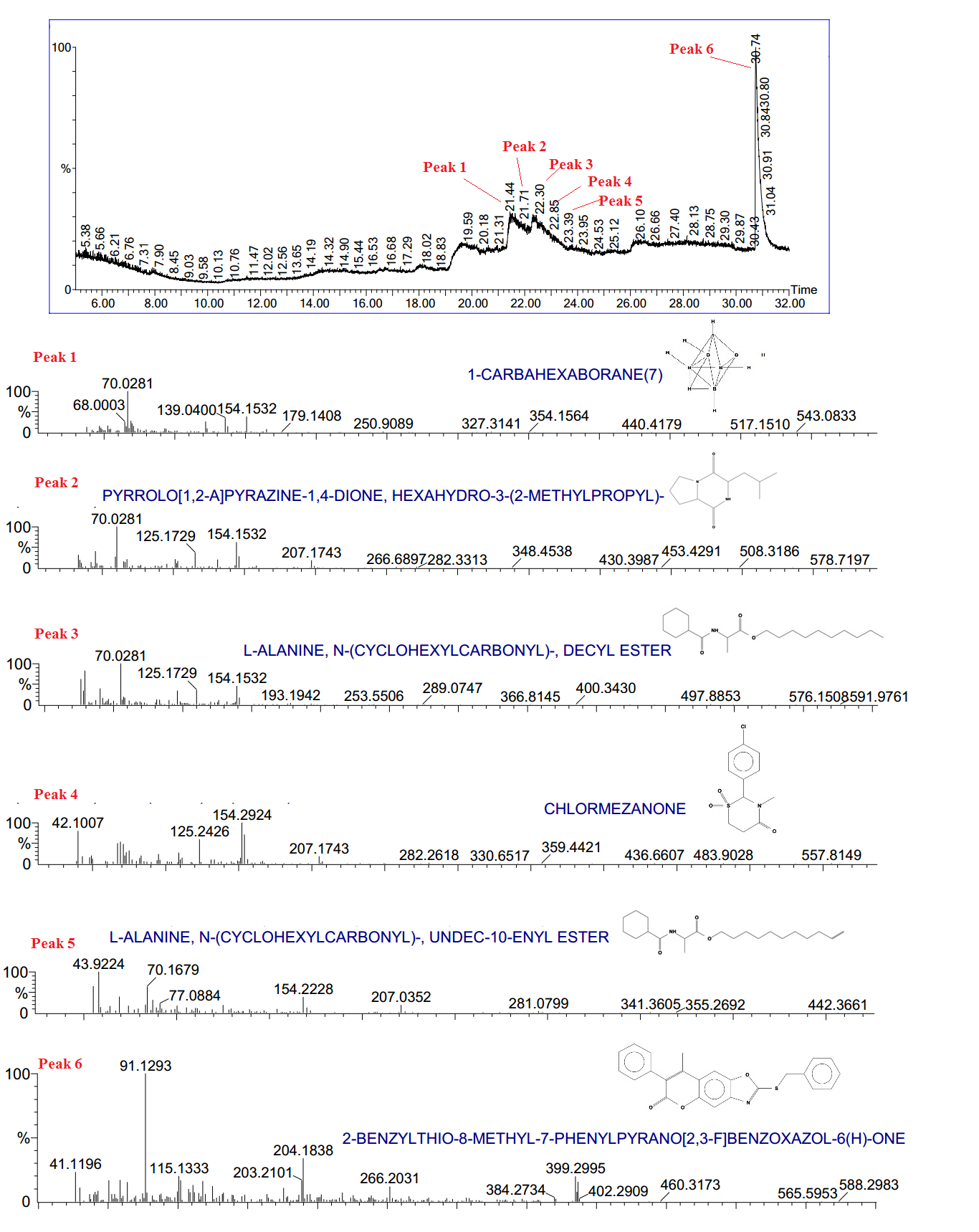


**Additional file 1: Fig.S7** VOCs determined using GC/MS in isolate *Streptomyces* sp. strain DST119

**
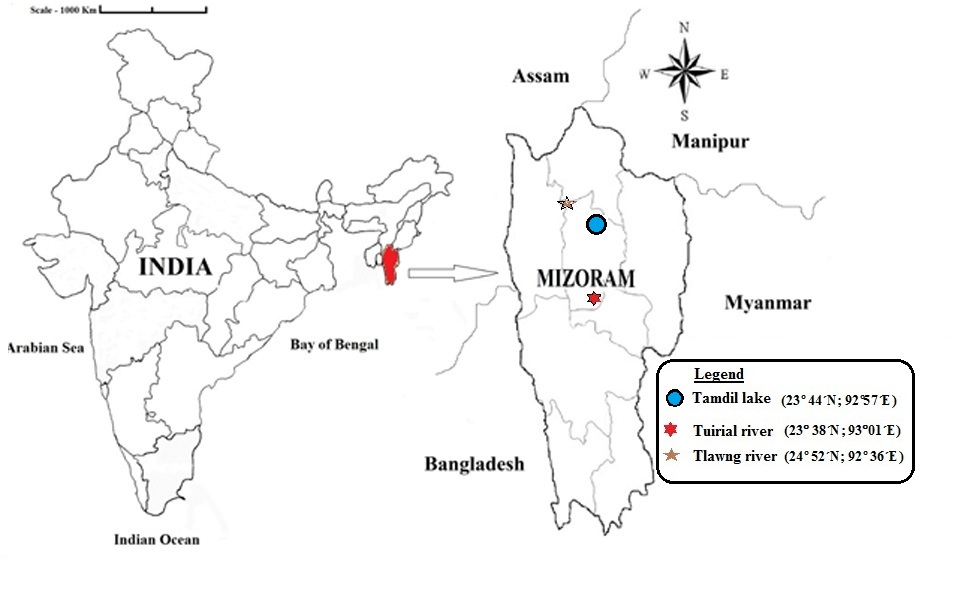
**

**Additional file 1: Fig.S8** Map showing the location of sampling site (Tamdil lake, Tlawng river and Tuirial river)
